# ATF6α inhibits ΔNp63α expression to promote breast cancer metastasis by the GRP78-AKT1-FOXO3a signaling

**DOI:** 10.1038/s41419-025-07619-8

**Published:** 2025-04-13

**Authors:** Hong Wang, Xin Yang, Liyuan Deng, Xuanyu Zhou, Jin Tao, Zhiqiang Wu, Hu Chen

**Affiliations:** 1 Department of Cardiothoracic Surgery, School of Clinical Medicine and The First Affiliated Hospital of Chengdu Medical College, Chengdu, China; 2 Department of Pediatrics, School of Clinical Medicine and The First Affiliated Hospital of Chengdu Medical College, Chengdu, China

**Keywords:** Metastasis, Cell migration

## Abstract

Endoplasmic reticulum (ER) stress is increasingly recognized as a driver of cancer progression; however, the precise molecular mechanisms by which ER stress facilitates tumor metastasis remain incompletely understood. In this study, we demonstrate that ER stress-activated ATF6α promotes breast cancer cell migration and metastasis by downregulating the expression of ΔNp63α, a key metastasis suppressor. Mechanistically, ATF6α reduces ΔNp63α expression through GRP78, which interacts with and activates AKT1. Activated AKT1 subsequently phosphorylates FOXO3a, leading to its degradation. Since FOXO3a directly transactivates ΔNp63α expression, its degradation results in reduced ΔNp63α levels. Furthermore, pharmacological inhibition or genetic knockdown of AKT1 upregulates ΔNp63α in vitro and suppresses tumor metastasis in vivo. Clinical analyses reveal that TP63 and FOXO3a expression are significantly reduced in breast cancer tissues compared to normal tissues, whereas ATF6 and GRP78 expression are elevated. Moreover, low TP63 and high GRP78 expression are associated with a poor prognosis in breast cancer patients. Collectively, these findings elucidate the pivotal role of the ATF6α-GRP78-AKT1-FOXO3a axis in chronic ER stress-mediated downregulation of ΔNp63α, establishing a molecular framework for targeting this pathway as a potential therapeutic strategy against breast cancer metastasis.

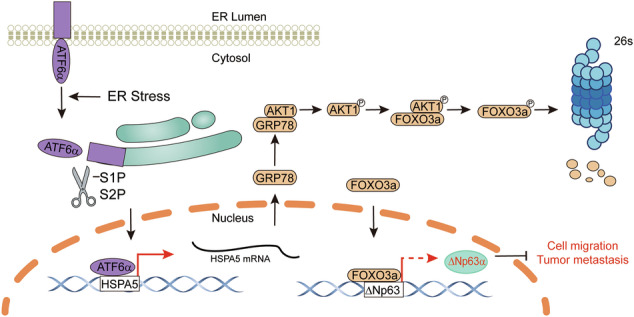

## Introduction

Tumor metastasis is the primary cause of cancer-related mortality [1]. Despite advances in cancer treatment, understanding and combating metastasis remains a critical challenge in oncology. The endoplasmic reticulum (ER) is responsible for protein synthesis, folding, and secretion [2]. Hostile environmental conditions, such as hypoxia, nutrient deprivation, oxidative stress, and cancer treatments, disrupt the ER protein-folding environment, and lead to misfolded/unfolded proteins accumulation in ER, referred to as ER stress [3]. ER stress activates a series of adaptive mechanisms that together are known as the unfolded protein response (UPR) [4]. The UPR comprise three parallel signalling branches: PERK-eIF2α, IRE1α-XBP1s and ATF6α [5]. In addition to induction of autophosphorylation and activation of IRE1 and PERK, ER stress also causes ATF6α transit through the Golgi complex, where it is activated through proteolytic cleavage by the enzymes S1P and S2P [6]. The cleaved N-terminal cytoplasmic domain of ATF6α is released from the Golgi complex, followed by translocation to the nucleus [5]. Acute ER stress can induce apoptosis, forming the foundation of several anticancer strategies [7]. Our previous studies demonstrated that ER stress triggers cancer cell apoptosis via PERK-ATF4 to activate TAp73α [8], a pro-apoptotic protein, which plays a pivotal role in promoting cancer cell death. In contrast, chronic ER stress fosters cell survival, proliferation, and metastasis [4]. However, the detailed mechanism of ER stress promotes tumor metastasis is largely unknown.

ΔNp63α, encoded by the TP63 gene, is the primary isoform of the p63 protein expressed in epithelial tissues and epithelial-derived tumors [9], and a master regulator in epidermal development [9, 10]. ΔNp63α is also a lineage-dependent oncogene in squamous cell carcinoma (SCC) and often as a diagnosis biomarker of SCCs, including head and neck, lung, and esophageal cancers [10, 11]. Besides, ΔNp63α could regulate cell adhesion and suppress metastatic progression [12, 13]. Acting as a transcription factor, ΔNp63α promotes the expression of various downstream effectors, including E-cadherin and Par3, which are critical for cell adhesion and epithelial integrity [14]. Our previous studies demonstrate that ΔNp63α can transactivate the expression of MKP3, CD82, or AMPK, and function as a critical metastatic suppressor [15–17]. ΔNp63α expression is controlled through transcription, post-transcription, and post-translational modifications, allowing it to respond to environmental [10]. In our previous work, we found that hypoxia-induced ER stress promotes breast cancer metastasis by downregulating ΔNp63α through two key signaling pathways: IRE1α-XBP1s and ATF6α [18]. However, the exact mechanism by which ATF6α suppresses ΔNp63α to facilitate tumor metastasis remains unknown and requires further exploration.

In this study, we demonstrate that chronic ER stress suppresses ΔNp63α expression, thereby promoting cell migration. Specifically, chronic ER stress activates ATF6α, which downregulates ΔNp63α expression through GRP78. GRP78 interacts with and activates AKT1, leading to the phosphorylation and subsequent degradation of FOXO3a. Since FOXO3a directly transactivates ΔNp63α expression; its degradation results in reduced ΔNp63α levels. Furthermore, pharmacological inhibition or genetic knockdown of AKT1 upregulates ΔNp63α in vitro and suppresses tumor metastasis in vivo. Collectively, these findings establish the ATF6α-GRP78-AKT1-FOXO3a axis as a molecular framework and highlight its potential as a therapeutic target for inhibiting breast cancer metastasis.

## Materials and Methods

### Cell culture and reagents

The MCF-10A, HCC1806, MDA-MB-231, and HEK-293T cell lines were sourced from the American Type Culture Collection (ATCC). MCF-10A cells were cultured in a 1:1 mixture of DMEM and F12 medium (Invitrogen, Carlsbad, CA, USA), supplemented with 5% horse serum (Invitrogen), 100 ng/mL cholera toxin (Sigma, St Louis, MO, USA), 10 μg/mL insulin (Sigma), 20 ng/mL epidermal growth factor (Invitrogen), and 500 ng/mL hydrocortisone (Sigma). MDA-MB-231 and HEK293FT cells were maintained in DMEM (Gibco, Rockville, MD, USA) with 10% FBS, while HCC1806 cells were cultured in RPMI-1640 (Gibco) with 10% FBS. All cell lines were mycoplasma-free and maintained with penicillin (100 U/mL)/streptomycin (100 μg/mL) at 37 °C in a humidified incubator with 5% CO_2_. Thapsigargin (TG), cycloheximide (CHX), MG-132, chloroquine (CQ), and MK2206 were obtained from Selleck (Selleck, Shanghai, China).

### Measuring in vitro guanine nucleotide exchange factor (GEF) activity of eIF2B

eIF2B enzymatic activity was assessed following established protocols [19]. Briefly, cells were washed with phosphate-buffered saline (PBS) and harvested via scraping in ice-cold homogenization buffer (45 mM HEPES-KOH, pH 7.4; 0.375 mM MgOAc; 75 mM EDTA; 95 mM KOAc; 10% glycerol; 1 mM DTT; 2.5 mg/mL digitonin) supplemented with EDTA-free protease inhibitors and PhosSTOP phosphatase inhibitors (Roche Applied Science). Lysates were processed through mechanical homogenization, and total protein concentration was determined using a bicinchoninic acid (BCA) assay. eIF2B activity was quantified by measuring the time-dependent exchange rate of [^3^H]GDP-bound eIF2α with unlabeled GDP.

### Plasmid construction

Short hairpin RNAs (shRNAs) targeting human ATF6α, GRP78, AKT1, or p63 were created by inserting specific oligonucleotides into a pLKO.1-puromycin lentiviral vector (10878, Addgene, Cambridge, MA, USA). The pLKO.1-scramble (#1864, Addgene) with non-targeting shRNA (shC) served as a negative control. The oligonucleotide sequences used are as follows: shATF6α #1: CTCGGTCAGTGGACTCTTA, shATF6α #2: ACAGAGTCTCTCAGGTTAAAT; shGRP78 #1: CTTGTTGGTGGCTCGACTCGA, shGRP78 #2: AGATTCAGCAACTGGTTAAAG; shAKT1 #1: CGAGTTTGAGTACCTGAAGCT; shAKT1 #2: GGACAAGGACGGGCACATTAA; shp63 #1: GAGTGGAATGACTTCAACTTT; shp63 #2: CATCTGACCTGGCATCTAATT.

The construct encoding the human ΔNp63α was previously described [18]. The open reading frames (ORFs) of the human ATF6α, GRP78, and FOXO3a, were obtained from the Miaoling Plasmid Sharing Platform (miaolingbio.com, Wuhan, Hubei, China) and cloned into the pLVX-puro lentiviral vector (632164, Clontech, Mountain View, CA, USA). Mutant constructs (ATF6α^R324C^ and FOXO3a^6A^) were generated using the KOD-Plus-Mutagenesis kit (SMK-101, Toyobo, Osaka, Japan). All constructs were verified by DNA sequencing.

For promoter activity assays, the fragment (−3000 bp to +1000) of the human ΔNp63 promoter was cloned into the Gluc-On promoter reporter vector (pEZX-PG04, GeneCopoeia, Guangzhou, China), referred to as ΔNp63-Gluc. Similarly, the fragment (−2000 to +1000) of the human HSPA5 promoter was inserted into the Gluc-On vector and designated as HSPA5-Gluc.

### Lentivirus packaging and infection

Recombinant lentiviral particles were produced by transfecting HEK-293T cells with the pMD2.G and psPAX2 packaging plasmids along with the appropriate backbone plasmid, using Lipofectamine 3000 (Invitrogen) following the manufacturer’s protocol. Viral supernatants were harvested 72 h post-transfection, filtered, and used to infect cells at approximately 50% confluence in the presence of 10 μg/mL polybrene. For selection of stable cell lines, cells were treated with 2 μg/mL puromycin (A1113803, Gibco) or 10 μg/mL blasticidin (A1113903, Gibco) at 48 h after infection.

### Gene deletion by CRISPR/Cas9 system

IRE1α knockout cells were generated through the CRISPR (clustered regularly interspaced short palindromic repeats)/Cas9 system. The guide sequence used was 5’- AGAGGACAGGCTCAATCAAA -3’ for human IRE1α, which was designed using the Optimized CRISPR Design at http://crispr.mit.edu, and cloned into the lentiCRISPR v2 vector (#52961, Addgene) for lentivirus packaging. HCC1806 cells were infected lentivrius expressing sgRNA targeting IRE1α, and selected with puromycin for stable cells. Next, cells were single-cell sorted by fluorescence-activated cell sorting (FACS) into 96-well plate. Knockout clones were selected by immunoblot analysis based on the lack of IRE1α proteins. IRE1α knockout cells were confirmed by DNA sequencing.

### Luciferase reporter assays

Luciferase reporter assays were conducted using the Secrete-PairTM Dual Luminescence Assay Kit (GeneCopoeia, USA) following the manufacturer’s protocol. In brief, cells were co-transfected with 500 ng of either the ΔNp63-Gluc reporter or the HSPA5-Gluc reporter, along with 750 ng of the ATF6α expression plasmids (ATF6α^WT^ or ATF6α^R324C^) or an empty vector (EV). Forty-eight hours after transfection, the culture media were collected, and both Gluc and SEAP activities were measured. ΔNp63-Gluc or HSPA5-Gluc activity was then normalized to SEAP activity.

### Quantitative RT-PCR

Quantitative RT-PCR (qPCR) was used to detect the mRNA levels as previously described [18]. qPCR primer sequences are listed below. GAPDH-F: AAGGTGAAGGTCGGAGTCAA, GAPDH-R: AATGAAGGGGTCATTGATGG; ΔNp63-F: GGAAAACAATGCCCAGACTC, ΔNp63-R: CTGCGCGTGGTCTGTGTTAT; FOXO3a-F: GTCTTCAGGTCCTCCTGTTCCT, FOXO3a-R: CACCAAAGAAGAGAGAAGGAGAGTT. CD82-F: CCATCAGGGTTCTCTTAGCAACT, CD82-F: CCAATTTTTCATACAGTTGCCCCT; MMP2-F: GCTTCCAAAGTAAACAGCAAGAGAA, MMP2-R: AACAGACTTAAAGAGGAAGCAAACC; ZEB1-F: ACCCTTGAAAGTGATCCAGC, ZEB1-R: CATTCCATTTTCTGTCTTCCGC.

### Immunoblot analysis and co-immunoprecipitation

For immunoblotting, cells were lysed in Beyotime lysis buffer (P0013, Beyotime, Shanghai, China) supplemented with protease (P1005, Beyotime) and phosphatase inhibitors (P1081, Beyotime). Equal amounts of protein lysates were separated by SDS-PAGE, transferred to PVDF membranes (Millipore, Darmstadt, Germany), and blocked with 5% nonfat milk in TBST. Membranes were incubated with the specified primary antibodies and HRP-conjugated secondary antibodies, followed by detection using enhanced chemiluminescence. Primary antibodies were obtained from the following sources: Cell Signaling Technology (Danvers, MA, USA) for Histone H3 (4499, 1:1000), H3K27me3 (9733, 1:1000), IRE1α (3294, 1:1000), pSTAT3 (9145, 1:1000), AKT1 (75692, 1:1000), pAKT1 (S473) (9018, 1:1000), FOXO3a (2497, 1:1000), pFOXO3a (Ser253) (13129, 1:1000), p21 (2947, 1:1000), and LC3 (2775, 1:1000); Abcam (Cambridge, MA, USA) for E-cadherin (ab40772, 1:1000) and ATF6 (ab122897, 1:500); BioLegend for XBP1s (619502, 1:500); ZEN-Bioscience (Chengdu, China) for p63 (381215, 1:1000); Millipore for Par3 (07-330, 1:1000); Abways Technology (Shanghai, China) for GAPDH (AB0036, 1:5000); Novus Biologicals for pIRE1α (NB100-2323, 1:1000); Proteintech for GRP78 (66574-1-Ig, 1:1000), E47 (21242-1-AP, 1:1000), and ubiquitin (10201-2-AP, 1:1000); and R&D for SOX2 (AF2018, 1:500).

For co-immunoprecipitation assays, cells were lysed in buffer containing 50 mM Tris-HCl (pH 7.4), 150 mM NaCl, 1 mM EDTA, and 1% NP-40. The lysates were then centrifuged at 15,000 g at 4 °C to remove debris. The resulting supernatants were incubated overnight at 4 °C on a rotator with either mouse anti-GRP78 (66574-1-Ig, Proteintech, 1: 50) or rabbit anti-AKT1 (75692, CST, 1: 50), normal mouse IgG (sc-2025, Santa Cruz, 1: 50) or normal rabbit IgG (2927, CST, 1:50) as a control. Protein A (sc-2001, Santa Cruz) or Protein G (sc-2002, Santa Cruz) agarose beads were subsequently added, followed by an additional 2-hour incubation at 4 °C. After four washes with PBS, the immunocomplexes were analyzed by immunoblotting.

### Chromatin immunoprecipitation (ChIP) assays

The JASPAR database (http://jaspar.genereg. net/) was used to predict potential transcription factor binding site on the human ΔNp63 gene promoter. Based on the predicted results from JASPAR, ChIP assays were used to confirm the binding regions of ATF6 or FOXO3a on the ΔNp63 gene promoter in HCC1806 cells with ChIP-IT Kit (53009, Active Motif, USA) using antibodies specific for ATF6 (ab122897, abcam, 1: 100), H3K27me3 (9733, CST, 1:50), H3K27ac (ab4729, abcam, 1:50); FOXO3a (2497, CST, 1: 100) and normal rabbit IgG (2927, CST, 1:50), as described previously [14]. Immunoprecipitated DNA was subjected to PCR or qPCR to amplify fragments of the ΔNp63 gene promoter elements using indicated primers listed in Supplementary Table 2.

### Immunofluorescent analyses

Immunofluorescent analysis was conducted as previously described [20]. In brief, cells cultured on coverslips were fixed with 4% paraformaldehyde in PBS, permeabilized with 0.1% Triton X-100, and blocked in 4% bovine serum albumin in PBS. Cells were incubated overnight at 4 °C with mouse anti-GRP78 (66574-1-Ig, Proteintech, 1: 100) and rabbit anti-AKT1 (75692, CST, 1: 100), followed by treatment with Rhodamine (TRITC)-conjugated donkey anti-mouse IgG (715-025-151, Jackson ImmunoResearch, PA, USA) or Fluorescein (FITC)-conjugated donkey anti-rabbit IgG (711-095-152, Jackson ImmunoResearch) for 1 h in the dark at room temperature. Nuclei were counterstained with DAPI for 5 min. Coverslips were mounted using Antifade Mounting Medium (P0126, Beyotime Biotechnology), and images were captured with a Leica TCS SP5 II microscope.

### Immunohistochemistry (IHC) staining

Human breast tumor tissue microarrays (OD-CT-RpBre03-002), comprising specimens from various breast cancer patients, were obtained from Shanghai Outdo Biotech (Shanghai, China). IHC analysis was conducted as previously described [18]. The microarrays included 31 breast cancer samples alongside matched adjacent non-tumor tissues and were used to assess the expression of ΔNp63 (619002, Biolegend, 1: 100), ATF6 (ab122897, abcam, 1: 100), GRP78 (66574-1-Ig, Proteintech, 1: 100), and FOXO3a (2497, CST, 1: 100). For quantitative analysis, slides were scanned with a NanoZoomer (Hamamatsu, Japan), and integrated optical density (IOD) measurements were performed using Image-Pro Plus 6.0. Average optical density (AOD) was calculated with the formula: AOD = IOD/Area [21].

### Transwell and wound-healing assays

Cells were suspended in serum-free medium and seeded into the inner chamber, while 600 μL of complete medium was added to the outer chamber. Cells were incubated for 24 hours at 37 °C in a humidified incubator with 5% CO_2_. After incubation, non-migratory cells on the inner side of the chamber were carefully removed by cotton swabs. Chambers were then stained with 0.1% crystal violet in 70% ethanol for 15 minutes and imaged under a light microscope (Nikon Eclipse Ti-S/L 100).

The wound-healing assay was mainly conducted as previously described [18]. Cells were grown to 90% confluency, then treated with Mitomycin C (1 μg/mL, S8146, Selleck, China) for 1 hour to exclude the impact of cell proliferation on wound-healing. Gently and slowly scratch the monolayer cell with a plastic pipette tip. After scratching, the cells were rinsed twice with PBS and incubated in medium containing 1% serum at 37 °C in a humidified incubator with 5% CO_2_. At specified time points, cells were imaged using a light microscope (Nikon Eclipse Ti-S/L 100), and wound closure was analyzed using ImageJ software.

### In vivo metastasis assay

Female nude mice, 6 weeks old (GemPharmatech Co., Ltd), were used for in vivo tumor metastasis experiments. To investigate the role of the ATF6α-ΔNp63α axis in metastasis, 18 mice were randomly assigned to three groups and injected via the tail vein with HCC1806 cells stably expressing either an empty vector (EV), ATF6α alone, or ATF6α together with ΔNp63α. To assess whether GRP78-induced metastasis depends on AKT1, another set of 18 mice received HCC1806 cells expressing EV or GRP78 through tail vein injection and were also randomized into three groups. Seven days post-inoculation, mice were treated by oral gavage with MK2206 (120 mg/kg daily for two weeks) or DMSO as a control. Mice were monitored daily and sacrificed 5 weeks after inoculation. Lungs were collected, fixed, paraffin-embedded, and sectioned for hematoxylin and eosin (H&E) staining, followed by histological analysis. Metastatic nodules in lung sections were quantified for each mouse.

### Human data from publicly available datasets

The Gene Expression Profiling Interactive Analysis 2 (GEPIA2) database (http://gepia2.cancer-pku.cn/) was used to evaluate mRNA expression levels of TP63, ATF6, GRP78, and FOXO3a. Kaplan–Meier survival curves of breast cancer (*n* = 1149) were generated using data from the KM Plotter database (www.kmplotter.com).

### Statistical analyses

Data from cell culture experiments were derived from three independent replicates and are presented as means ± SD. Unless otherwise specified, the differences between groups were evaluated by using a two-tailed unpaired Student’s t-test when only two groups were compared or by using ANOVA when more than two groups were compared. The homogeneity of clinical data variances was assessed with Levene’s test in SPSS 16 software prior to analysis. Statistical significance was set at *P* < 0.05.

## Results

### Chronic ER stress promotes cell migration via downregulating of ΔNp63α expression

**Fig. 1:**
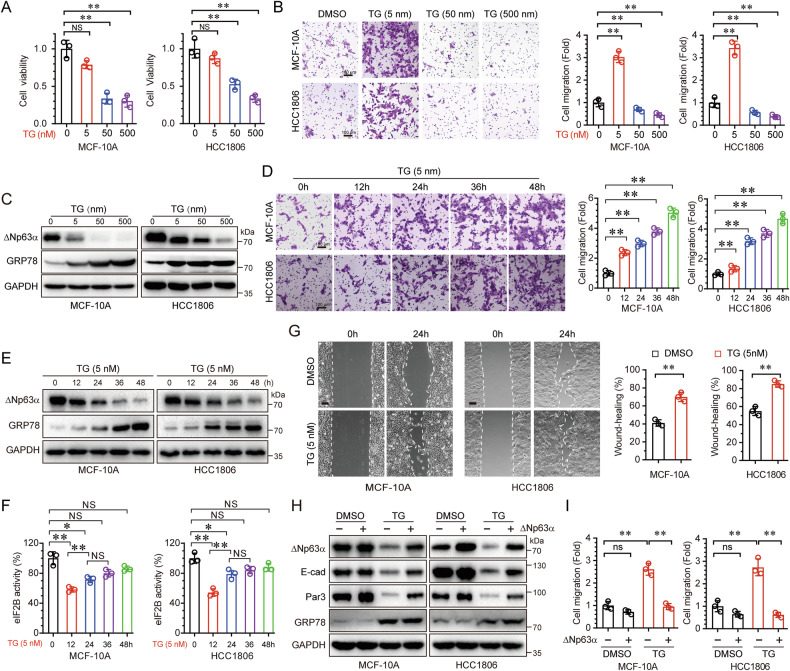
ER stress promotes cell migration through downregulation of ΔNp63α. **A**–**C** MCF-10A or HCC1806 cells were treated with thapsigargin (TG) at the indicated concentrations for 24 h, followed by CCK8 assays to assess cell viability (**A**) transwell assays to evaluate cell migration (**B**) and immunoblotting to measure protein levels (**C**). **D**–**F** Cells treated with 5 nM TG for the indicated times were analyzed by transwell assays (**D**) immunoblotting (**E**) eIF2B GEF activity assays (**F**). **G** MCF-10A or HCC1806 cells treated with 5 nM TG for 24 h were subjected to wound-healing assays to assess cell migration. **H**, **I** MCF-10A or HCC1806 cells stably expressing ΔNp63α or an empty vector were treated with 5 nM TG for 24 h and analyzed by immunoblotting (**H**) or transwell migration assays (**I**). Results are presented as means ± SD from three independent experiments performed in triplicates. Scale bar = 100 μm, *P < 0.05, **P < 0.01, NS no significance.

Endoplasmic reticulum (ER) stress has been well-documented to significantly influence cancer cell proliferation and malignancy [3, 22]. To investigate the effects of ER stress on cell viability and migration, we utilized thapsigargin (TG), a known inhibitor of the sarco/endoplasmic reticulum Ca²⁺-ATPase (SERCA) pump [23], to induce ER stress and evaluate its impact on these cellular processes. As shown in Fig. 1, the viability of both immortalized human breast epithelial MCF-10A cells and triple-negative breast cancer (TNBC) HCC1806 cells progressively decreased with the increase of TG concentrations (Fig. 1A). Notably, low-dose TG (5 nM), which had minimal effects on cell viability, significantly promoted cell migration (Fig. 1B). ΔNp63α, a critical tumor metastasis suppressor, plays a pivotal role in various signaling pathways, including oncogenic activation, Hippo signaling, and hypoxia response [14, 18, 24]. However, the role of ΔNp63α in ER stress-mediated cell migration or tumor metastasis remains poorly understood. As illustrated in Fig. 1C, ΔNp63α protein levels decreased progressively with the increase of TG concentrations, suggesting that ER stress promotes cell migration by downregulating ΔNp63α. Given the chronic stress contributes to ER dysfunction and various pathological conditions, we investigated its role in cell migration by treating cells with TG (5 nm) for 0-48 hours to monitor temporal changes. As expected, low-dose TG induced chronic ER stress accelerated cell migration in MCF-10A and HCC1806 cells (Fig. 1D), concomitant with a gradual increase of GRP78 (an ER stress marker) and a decrease of ΔNp63α (Fig. 1E). We next assessed eIF2B activity by measuring its guanine nucleotide exchange factor (GEF) function in TG-treated and control cells. eIF2B activity decreased after 12 hours of TG treatment but was restored as the treatment duration extended (Fig. 1F), indicating that the cells may activate an adaptive response to promote cell migration. Consistent with this, low-dose TG treatment enhanced cell mobility, as demonstrated by wound-healing assays (Fig. 1G). Importantly, restoring ΔNp63α expression reinstated the expression of E-cadherin and Par3, thereby fully rescuing ER stress-induced cell migration (Fig. 1H, I). These findings suggest that the downregulation of ΔNp63α is a central mechanism underlying ER stress-induced cell motility.

### ER stress suppresses ΔNp63α and promotes cell migration through activation of ATF6α

**Fig. 2:**
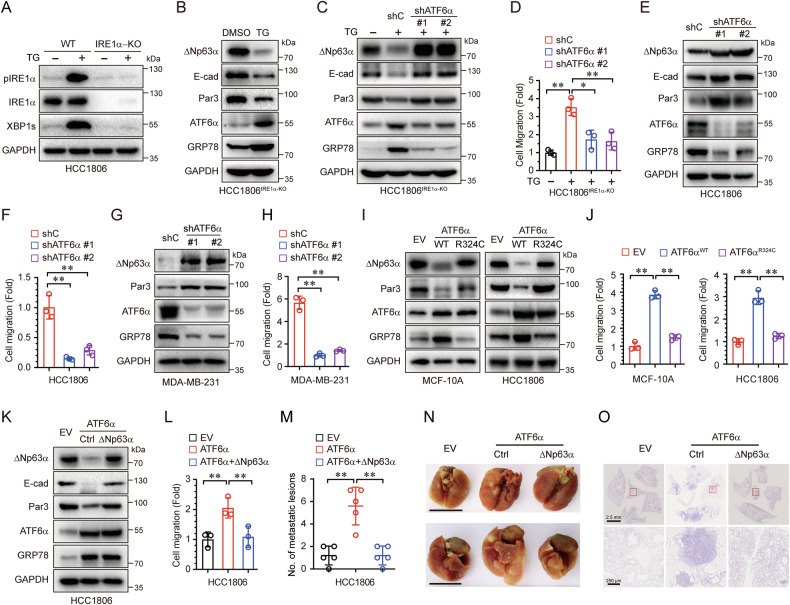
ER stress suppresses ΔNp63α and promotes cell migration through activation of ATF6α. **A** HCC1806 cells (WT or IRE1α-KO) were treated with TG (5 nM) for 24 hours, and analyzed by immunoblotting. **B** IRE1α-KO HCC1806 cells were treated with TG (5 nM) for 24 h and subjected to immunoblot. **C**, **D** IRE1α-KO HCC1806 cells stably expressing either control shRNA (shC) or ATF6α-specific shRNAs (shATF6α-#1 and shATF6α-#2) were treated with TG (5 nM) for 24 h and analyzed by immunoblotting (**C**) and transwell assays (**D**). **E**–**H** HCC1806 or MDA-MB-231 cells stably expressing either control shRNA (shC) or ATF6α-specific shRNAs (shATF6α-#1 and shATF6α-#2) were treated with TG (5 nM) for 24 h and analyzed by immunoblotting (**E**, **G**) and transwell assays (**F**, **H**). **I**, **J** MCF-10A or HCC1806 cells stably expressing an empty vector (EV) or ATF6α (WT or R324C) were analyzed by immunoblotting (**I**) and transwell assays (**J**). **K**–**O** HCC1806 cells stably expressing EV, ATF6α, or ATF6α-ΔNp63α were analyzed by immunoblotting (**K**) and transwell assays (**L**). HCC1806 stable cells were injected into the tail vein of nude mice (6 per group). After 50 days, lungs were collected, and metastatic nodules on the surface were quantified (**M**, **N**). Lung tissues were subjected to H&E staining for histological evaluation (**O**). Results are presented as means ± SD from three independent experiments performed in triplicates. **P < 0.01, NS: no significance.

Our previous study demonstrated that hypoxia-induced ER stress downregulated ΔNp63α through the IRE1α-XBP1s and ATF6α pathways [18]. However, the precise mechanism by which ATF6α downregulates ΔNp63α remains unknown. To investigate whether ER stress downregulates ΔNp63α via ATF6α, we first generated IRE1α-knockout (IRE1α-KO) cells using CRISPR-Cas9 technology. As shown in Supplementary Fig. 1A, a single nucleotide deletion in the coding sequence of the ERN1 gene in IRE1α-KO cells introduced a premature stop codon, resulting in the termination of IRE1α protein translation (Supplementary Fig. 1B). Notably, XBP1s, the downstream target of IRE1α, was not activated following TG treatment in IRE1α-KO cells (Fig. 2A). These results confirm that IRE1α was successfully knocked out in HCC1806 cells. Interestingly, TG treatment still downregulated ΔNp63α and its downstream targets, E-cadherin and Par3, in IRE1α-KO cells (Fig. 2B), suggesting that ER stress-mediated ΔNp63α downregulation may occur through alternative pathways independent of IRE1α. To further explore whether ATF6α suppresses ΔNp63α to promote cell migration, we performed ATF6α knockdown in IRE1α-KO HCC1806 cells. As shown in Fig. 2C, ATF6α knockdown significantly restored ΔNp63α protein expression, which had been downregulated by TG treatment. Consequently, ATF6α knockdown markedly suppressed chronic ER stress-induced cell migration (Fig. 2D). Moreover, ATF6α knockdown in both HCC1806 and MDA-MB-231 cells led to the upregulation of ΔNp63α protein expression and inhibition of cell migration (Fig. 2E–H). In contrast, ectopic expression of wild-type ATF6α, but not the transcription-defective mutant (R324), significantly downregulated ΔNp63α expression and promoted cell migration in both MCF-10A and HCC1806 cells (Fig. 2I, J). These findings demonstrate that ATF6α-mediated suppression of ΔNp63α expression and promotion of cell migration are dependent on its transcriptional activity.

We next investigated the causal role of ΔNp63α in the biological effects of ATF6α-induced cell motility. As shown in Fig. 2K, L, ectopic expression of ATF6α significantly reduced ΔNp63α expression (Fig. 2K), concomitant with enhanced cell migration (Fig. 2L). This increase in cell migration was effectively reversed by restoring ΔNp63α expression, which also reinstated the expression of E-cadherin and Par3 expression, key downstream effectors of ΔNp63α (Fig. 2K, L). To further explore the role of the ATF6α-ΔNp63α axis in tumor metastasis, we utilized a tail vein injection mouse metastasis model. As shown in Fig. 2M–O, mice injected with HCC1806 cells overexpressing ATF6α developed multiple metastatic nodules on the lung surfaces, an effect that was significantly attenuated by simultaneous overexpression of ΔNp63α. These findings indicate that ATF6α-mediated suppression of ΔNp63α is critical for promoting tumor metastasis. Collectively, these results demonstrate that chronic ER stress downregulates ΔNp63α and promotes cell migration through ATF6α activation.

### ATF6α suppresses ΔNp63α and promotes cell migration through GRP78

**Fig. 3:**
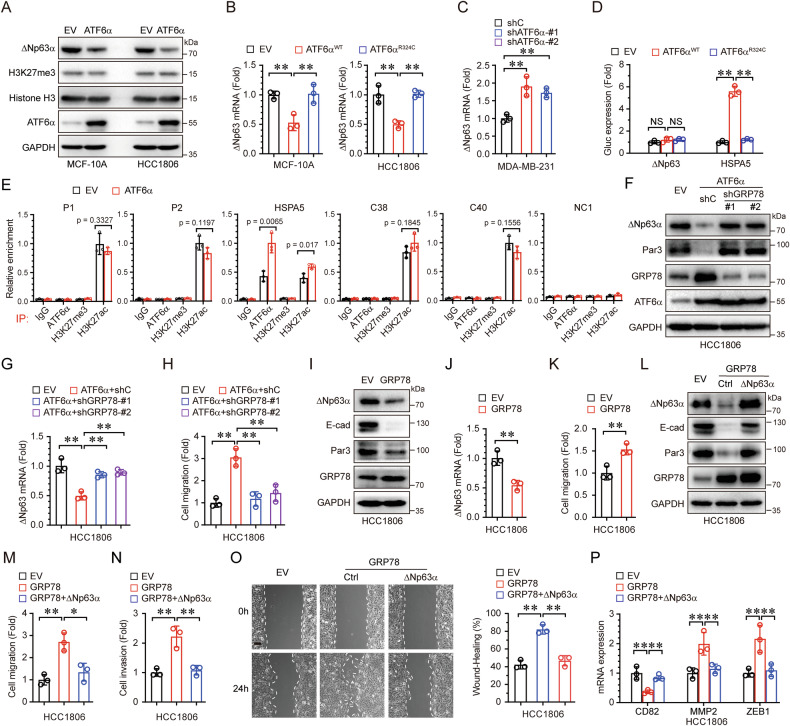
ATF6α suppresses ΔNp63α and promotes cell migration through GRP78. **A** MCF10A or HCC1806 cells stably expressing ATF6α or an empty vector (EV) were analyzed by immunoblotting. **B** MCF-10A or HCC1806 cells stably expressing EV or ATF6α (WT or R324C) were subjected to qPCR assays. **C** MDA-MB-231 cells stably expressing control shRNA (shC) or ATF6α-specific shRNAs (shATF6α-#1 and shATF6α-#2) were analyzed by qPCR. **D** HEK-293T cells were co-transfected with ΔNp63-Gluc or HSPA5-Gluc reporter and ATF6α (WT or R324C) expression plasmids. Gluc and SEAP activities in the medium were measured 48 h post-transfection. **E** ChIP assays were performed in HCC1806 cells (EV or ATF6α) using indicated antibodies or a normal rabbit IgG. Primers specific for P1, P2, HSPA5, C38 or C40 were used for qPCR; a randomly chosen segment (−516 to −314) served as a negative control (NC1). **F**–**H** HCC1806 cells stably expressing ATF6α were infected with shRNAs targeting GRP78 and subsequently analyzed by immunoblotting (**F**) qPCR (**G**) and transwell assays (**H**). **I**–**K** HCC1806 cells stably expressing GRP78 or EV were analyzed by immunoblotting (**I**), qPCR (**J**), and transwell assays (**K**). **L**–**P** HCC1806 cells stably expressing GRP78 were infected with lentivirus expressing ΔNp63α and subsequently subjected to immunoblotting (**L**) transwell for migration (**M**) and invasion (**N**) wound-healing (**O**) and qPCR assays (**P**). Results are presented as means ± SD from three independent experiments performed in triplicates. Scale bar = 100 μm, **P < 0.01, NS: no significance.

Our previous study demonstrated that hypoxia-induced ER stress suppresses ΔNp63α via alterations in the H3K27me3 mark [18]. However, ectopic expression of ATF6α had minimal effect on H3K27me3 levels in MCF-10A or HCC1806 cells (Fig. 3A), suggesting that ATF6α reduces ΔNp63α expression via a H3K27me3-independent mechanism. Ectopic expression of wild-type ATF6α, but not R324C mutant, significantly downregulated ΔNp63α mRNA levels in MCF-10A and HCC1806 cells (Fig. 3B). Conversely, knockdown of ATF6α significantly upregulated ΔNp63α mRNA levels in MDA-MB-231 cells (Fig. 3C). These results indicate that ATF6α downregulates ΔNp63α expression at the transcriptional level. To determine whether ATF6α directly regulates ΔNp63α expression, we conducted luciferase reporter assays. As shown in Fig. 3D, wild-type ATF6α, but not R324C mutant, increased the reporter activity of HSPA5 (which encodes GRP78), a well-known ATF6α target. However, neither the wild-type ATF6α nor the R324C mutant activated the ΔNp63α reporter. Additionally, we analyzed the ΔNp63 gene promoter for putative transcription factors using the KnockTF database (http://www.licpathway.net/KnockTFv2/index.php), and found that ATF6α was not a transcription factor for ΔNp63 (Supplementary Table 1). We also analyzed the JASPAR database (https://jaspar.elixir.no/) and identified two putative ATF6α binding elements, termed P1 and P2, on the ΔNp63 gene promoter (Supplementary Fig. 2). As expected, ChIP assays confirmed that ATF6α can bind to HSAP5 promoter, and ectopic expression of ATF6α enhanced this binding (Fig. 3E and Supplementary Fig. 3). However, ATF6α did not bind to the P1 or P2 sites, nor to the C38 and C40 elements, which are known enhancers of ΔNp63 gene [25]. Furthermore, ATF6α did not alter the H3K27ac or H3K27me3 marks at the P1, P2, C38, and C40 sites of the ΔNp63 gene promoter (Fig. 3E), indicating that ATF6α suppresses ΔNp63α expression in a H3K27me3-independent manner. Taken together, these results suggest that ATF6α likely does not directly repress ΔNp63α transcription.

GRP78 is a well-documented target of ATF6α and plays a crucial role in tumor metastasis [26]. To investigate whether GRP78 contributes to ATF6α-mediated ΔNp63α downregulation and cell migration, we performed GRP78 knockdown in ATF6α-overexpressing HCC1806 cells. As shown in Fig. 3F, G, GRP78 knockdown significantly unregulated both the protein and mRNA levels of ΔNp63α, which had been suppressed by ATF6α. Consequently, GRP78 knockdown dramatically suppressed ATF6α-mediated cell migration (Fig. 3H). Furthermore, ectopic expression of GRP78 significantly reduced ΔNp63α protein and mRNA levels (Fig. 3I, J) and promoted cell migration (Fig. 3K). To further explored the role of ΔNp63α in GRP78-induced cell motility, we examined the effects of GRP78 overexpression. Ectopic expression of GRP78 again led to a significant reduction in ΔNp63α expression (Fig. 3L), which was accompanied by increased cell migration and invasion (Fig. 3M–O). This effect was effectively reversed by restoring ΔNp63α expression, which also reinstated the expression of key downstream effectors of ΔNp63α, including E-cadherin, Par3, CD82, MMP2, and ZEB1 [16, 27–29] (Fig. 3L, P). In summary, these findings indicate that ER stress-activated ATF6α suppresses ΔNp63α and promotes cell migration via GRP78.

### GRP78 interacts with AKT1 to inhibit ΔNp63α by downregulating FOXO3a

**Fig. 4:**
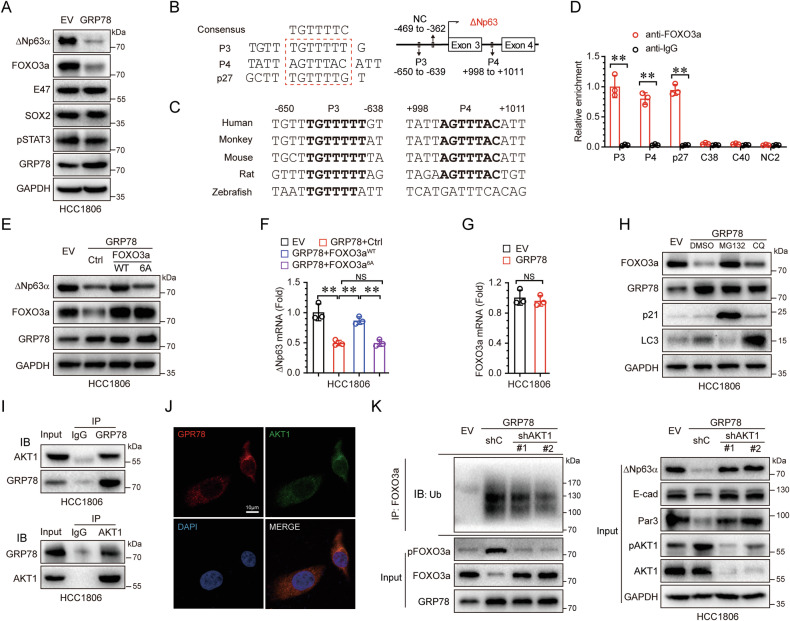
GRP78 interacts with AKT1 to inhibit ΔNp63α by downregulating FOXO3a. **A** HCC1806 cells stably expressing GRP78 were analyzed by immunoblotting. **B**, **C** Two putative FOXO3a-binding elements (P3: −650 to −639; P4: +998 to +1011) on the ΔNp63 gene promoter and the FOXO3a-binding consensus sequence are illustrated (**B**) the P3 and P4 sequences of the p63 gene were aligned (**C**). **D** ChIP assays were performed in HCC1806 cells using a FOXO3a-specific antibody or a control rabbit normal IgG; a randomly chosen segment (−469 to −362) served as a negative control (NC2). **E**, **F** HCC1806 cells stably expressing GRP78 were infected with lentivirus expressing either wild-type FOXO3a (WT) or mutant FOXO3a (6 A) and analyzed by immunoblotting (**E**) and qPCR (**F**). **G** qPCR assays were performed on HCC1806 cells stably expressing GRP78. **H** HCC1806 cells stably expressing GRP78 were treated with DMSO, MG132 (10 μM), or CQ (20 μM) for 24 h and subsequently analyzed by immunoblotting. **I** HCC1806 cells were subjected to immunoprecipitation (IP) using anti-GRP78 or anti-AKT1 antibodies, and co-precipitating endogenous AKT1 or GRP78 was detected by immunoblotting (IB). **J** HCC1806 cells were analyzed by immunofluorescence (IF) to assess GRP78 and AKT1 localization. **K** HCC1806 cells stably expressing GRP78 and infected with AKT1-specific shRNAs were subjected to immunoprecipitation with anti-FOXO3a, and co-precipitating ubiquitin was detected by immunoblotting. Results are presented as means ± SD from three independent experiments performed in triplicates. **P < 0.01, NS: no significance.

We next explored the molecular mechanism by which GRP78 regulates ΔNp63α gene transcription in response to ER stress. First, we examined whether GRP78 modulates the activity of known transcription factors that transactivate ΔNp63α expression, including FOXO3a [14], E47 [30], SOX2 [31], and STAT3 [32]. As shown in Fig. 4A, ectopic expression of GRP78 only significantly downregulated FOXO3a expression, which coincided with a decrease of ΔNp63α levels. To determine whether FOXO3a directly regulates ΔNp63α expression, we performed computational analysis of the ΔNp63 gene promoter sequence by the JASPAR database. As shown in Fig. 4B, this analysis revealed two putative FOXO3a binding sites: the first site (P3: −650 to −639 relative to the ΔNp63 transcriptional start site) and the second site (P4: +998 to +1011). Both sites contain sequences matching the FOXO3a consensus binding sites (TTGTTTTC) [33], which is also found in the documented FOXO3a binding site on the p27 promoter [34]. Additionally, P3 is highly conserved across five vertebrate species, while P4 is conserved in four mammalian species (Fig. 4C). ChIP assays confirmed that FOXO3a bind to the P3 and P4 sites of the ΔNp63 gene promoter (Fig. 4D and Supplementary Fig. 4E), but not to the C38, C40 elements. These results demonstrate that FOXO3α directly transactivates ΔNp63α expression.

To determine whether FOXO3a plays a key role in GRP78-mediated ΔNp63α downregulation, we restored FOXO3a expression in GRP78-overexpressing HCC1806 cells. As shown in Fig. 4E, F, restoration of wild-type FOXO3a, but not the mutant FOXO3a^6A^, in which Thr^179^, Ser^399^, Ser^413^, Ser^555^, Ser^588^, and Ser^626^ were replaced by alanine, significantly upregulated ΔNp63α protein and mRNA levels, both of which had been downregulated by GRP78. Notably, ectopic expression of GRP78 had little effect on FOXO3a mRNA levels (Fig. 4G), indicating that GRP78 downregulates FOXO3a expression at the post-transcriptional level. To further investigate how GRP78 affects FOXO3a protein levels, we treated GRP78-overexpressing HCC1806 cells with either a proteasome inhibitor (MG132) or a lysosome inhibitor (chloroquine, CQ). Only MG132 blocked FOXO3a degradation (Fig. 4H), suggesting that GRP78 promotes FOXO3a degradation via the proteasome pathway. Our previous study demonstrated that oncogenic signals downregulate FOXO3a through AKT1 [14]. Using the BioGRID protein-protein interaction database, we identified a potential interaction between GRP78 and AKT1. This interaction was confirmed in HCC1806 cells by co-immunoprecipitation analysis using anti-GRP78 and anti-AKT1 antibodies (Fig. 4I). Immunofluorescence assays revealed that GRP78 and AKT1 predominantly co-localize in the cytoplasm (Fig. 4J). Furthermore, immunoprecipitation assays showed that the levels of ubiquitin-conjugated FOXO3a were increased in GRP78-overexpressing cells (Fig. 4K). However, knockdown of AKT1 reduced the levels of ubiquitin-conjugated FOXO3a induced by GRP78, significantly elevating FOXO3a protein levels along with ΔNp63α and its targets (E-cadherin and Par3). In summary, these results demonstrate that GRP78 suppresses ΔNp63α expression by interacting with AKT1 to promote FOXO3a degradation.

### Inhibition of AKT1 restores GRP78-mediated cell migration and tumor metastasis

**Fig. 5:**
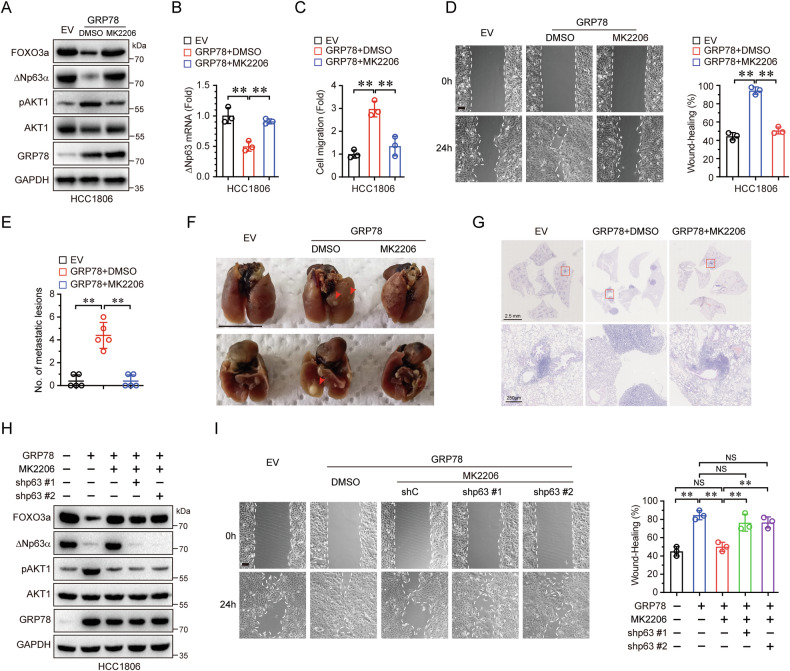
Inhibition of AKT1 reverses GRP78-mediated cell migration and tumor metastasis. **A**–**D** HCC1806 cells stably expressing GRP78 were treated with DMSO or MK2206 (5 μM) for 24 h and subsequently analyzed by immunoblotting (**A**) qPCR (**B**) transwell (**C**) and wound-healing assays (**D**), Scale bar = 100 μm. **E**–**G** HCC1806 cells stably expressing GRP78 were injected into the tail veins of nude mice (5 per group). Seven days post-inoculation, mice received daily treatments of either MK2206 (120 mg/kg) or DMSO for 14 days. On day 50 post-inoculation, lungs were collected, and surface metastatic nodules were quantified (**E**, **F**). Lung tissues were processed for H&E staining for histological evaluation (**G**). **H**, **I** HCC1806 cells stably expressing GRP78 were infected with lentiviral shRNAs targeting p63 (shp63 #1 or shp63 #2) and treated with DMSO or MK2206 (5 μM) for 24 h, followed by immunoblotting (**H**) wound-healing assays (**I**), Scale bar = 100 μm. Results are presented as means ± SD from three independent experiments performed in triplicates. **P < 0.01, NS no significance.

To determine whether GRP78-AKT1-mediated downregulation of FOXO3a is responsible for ΔNp63α transcription and cell migration, we treated GRP78-overexpressing HCC1806 cells with MK2206, a phase II AKT inhibitor used in breast cancer treatment. As shown in Fig. 5A, inhibition of AKT activity by MK2206 significantly reduced AKT1 phosphorylation and restored FOXO3a protein levels, which had been suppressed by GRP78. This restoration led to the recovery of both ΔNp63α protein and mRNA expression (Fig. 5A, B), and, notably, inhibited GRP78-mediated cell migration (Fig. 5C, D). To further explored the role of the GRP78-AKT1 axis in tumor metastasis, we employed a tail vein injection mouse metastasis model. As shown in Fig. 5E–G, mice injected with GRP78-overexpressing HCC1806 cells developed multiple metastatic nodules on the lung surfaces, an effectively attenuated by MK2206 treatment, indicating that AKT1 activity is critically for GRP78-mediated tumor metastasis. To investigate whether the upregulation of ΔNp63α by AKT1 inhibition contributes to the suppression of cell migration, we knocked down ΔNp63α in GRP78-stably expressed HCC1806 cells. As shown in Fig. 5H, I, ectopic expression of GRP78 significantly reduced ΔNp63α expression and promoted cell migration; however, inhibition of AKT1 markedly restored ΔNp63α expression and suppressed cell migration; Conversely, knockdown of ΔNp63α expression significantly promoted cell migration. Collectively, these findings demonstrate that inhibiting AKT1 reverses GRP78-mediated cell migration and tumor metastasis by upregulating ΔNp63α.

### Opposing expression patterns of TP63 and ATF6α/GRP78 in breast cancer

**Fig. 6:**
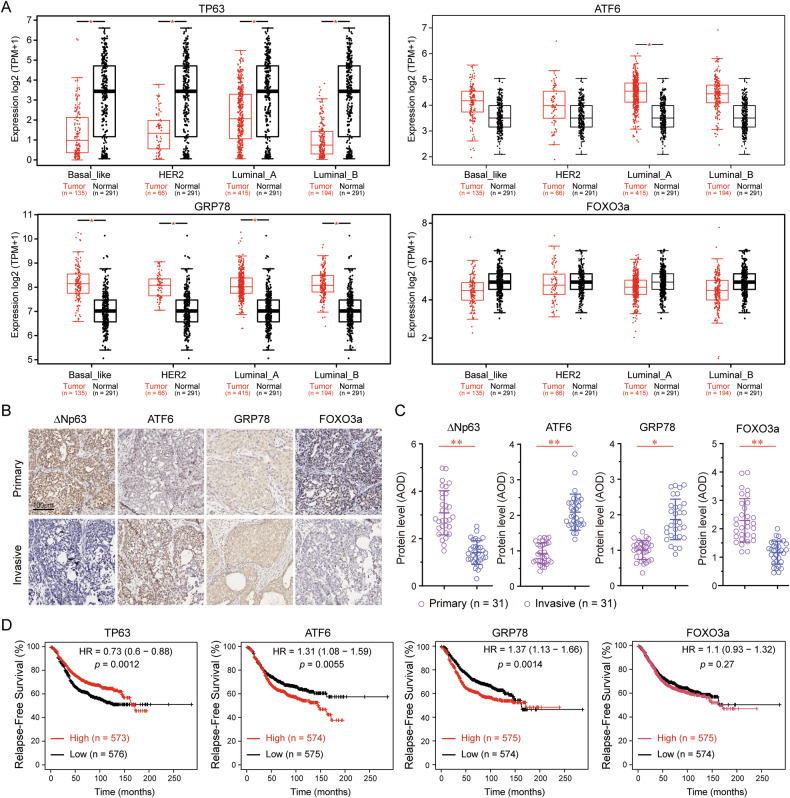
Opposing expression trends of TP63 and ATF6α/GRP78 in breast cancer. **A** GEPIA 2 datasets were analyzed to compare mRNA expression levels of TP63, ATF6, GRP78 and FOXO3a in human normal breast tissue and breast carcinoma. **B**, **C** Tissue microarray slides containing sequential sections of human breast carcinoma samples were subjected to IHC staining (**B**) and quantitative analysis (AOD) to evaluate protein expression levels of ΔNp63, ATF6, GRP78, and FOXO3a (**C**). **D** Kaplan-Meier datasets were used to assess patient survival, with cases stratified based on TP63, ATF6, GRP78, or FOXO3a mRNA expression levels. *P < 0.05, **P < 0.01, NS: no significance.

To compare the expression levels of ΔNp63α, ATF6α, GRP78, and FOXO3a in breast cancer tissue versus normal tissue, we analyzed publicly available datasets. In the GEPIA database, TP63 gene expression was significantly lower in breast cancer tissues, including basal-like, HER2-positive, luminal A and luminal B subtypes, compared to normal tissues (Fig. 6A). Similarly, FOXO3a expression was downregulated in breast cancer tissues compared to normal tissues. In contrast, the expression of ATF6 or GRP78 was significantly elevated in breast cancer tissues compared to normal tissues. Consistent with these findings, immunohistochemical (IHC) analyses of human breast cancer biopsy samples revealed that ΔNp63α and FOXO3a protein levels were significantly decreased in invasive breast cancer specimens compared to primary breast cancer specimens, whereas ATF6 and GRP78 protein levels were markedly increased in invasive breast cancer specimens (Fig. 6B, C). Moreover, low TP63 expression (*p* = 0.0012), high ATF6 expression (*p* = 0.0055) and high GRP78 expression (*p* = 0.0014) were significantly associated with poor prognosis in breast cancer patients (Fig. 6D).

## Discussion

In tumors, chronic ER stress can trigger adaptative mechanisms that promote metastasis. Key signaling pathways within the UPR have been shown to regulate various aspects of metastasis, including cell survival, migration, invasion, and treatment resistance. IRE1α activation leads to the splicing of XBP1s mRNA, producing XBP1s, which upregulates genes involved in survival and adaptation, thereby enhancing cancer cell invasiveness [35]. Recently, our research demonstrated that hypoxia inhibits ΔNp63α expression to promote tumor metastasis through IRE1α-XBP1s pathway [18]. PERK signaling has been linked to metastatic behaviors through mediators, such as LAMP3 and CREB3L1 [36, 37]. Additionally, ATF6α has been reported to promote tumor metastasis through ENTPD5 [38]. In this study, we provide evidence that ATF6α suppresses ΔNp63α through the GRP78-AKT1-FOXO3a axis to facilitate breast cancer metastasis.

ATF6α is a key sensor in the UPR, playing a central role in cellular adaptation to ER stress. Pharmacological activation of ATF6 has been shown to confer global protection in diverse disease models by reprograming cellular proteostasis [39]. ATF6α also contributes to myocardial adaptation to chronic hypoxia [40] and reduces myocardial ischemia/reperfusion injury [41]. Additionally, ATF6 safeguards organelle homeostasis and mitigates cellular aging in human mesenchymal stem cells [42]. In the context of cancer, ATF6 plays a dual role in promoting tumor growth and metastasis. While ATF6 activation initially supports cell survival during early tumor development by alleviating ER stress [43], its sustained activation has been linked to tumor progression and metastasis. Our previous findings demonstrated that hypoxia-induced ER stress downregulates ΔNp63α via the ATF6α pathway, facilitating metastatic spread, particularly in breast cancer models [18]. In this study, we elucidate the detailed mechanism by which ATF6α suppresses ΔNp63α transcription through the GRP78-AKT1-FOXO3a axis. A genome-wide CRISPR/Cas9 screen identified calreticulin as a selective repressor of ATF6α [44]. Given its ability to modulate both cell survival and metastatic behaviors, ATF6 represents an attractive therapeutic target for limiting cancer progression.

GRP78, also known as BiP, is a central molecular chaperone within the ER and a key marker of ER stress [45]. As a master regulator of the UPR, GRP78 plays a critical role in maintaining protein homeostasis under stressful conditions by promoting proper protein folding, preventing aggregation, and facilitating the degradation of misfolded protein [46]. During tumor growth, where nutrient and oxygen deprivation are common, GRP78 is upregulated to meet the increased protein-folding demands, enabling cancer cells to adapt and survive under adverse conditions [47]. GRP78 haploinsufficiency has been shown to suppress acinar-to-ductal metaplasia in mutant Kras-driven pancreatic tumorigenesis [48]. GRP78 also plays a significant role in promoting tumor growth and metastasis [49], including through interactions with CRIPTO or CDK7 [26, 50]. In this study, we found that GRP78 triggers breast cancer metastasis by downregulating ΔNp63α expression. Furthermore, we demonstrate that GRP78 interacts with AKT1, leading to the phosphorylation and degradation of FOXO3a, a transcription factor that activates ΔNp63α expression. Hyperactivation of AKT1 is observed in over 50% of human tumors [51], and our previous study demonstrated that AKT1 plays a central role in transducing oncogenic signals (including, PI3K, Ras and Her2) to promote tumor metastasis by suppressing ΔNp63α expression [14]. In this study, pharmacologic inhibition AKT1 reversed GRP78-mediated downregulation of FOXO3a and ΔNp63α expression, thereby inhibiting cell migration and tumor metastasis. Indeed, FOXO3a has been widely implicated in metastasis inhibition [52, 53], suggesting that targeting AKT1 inhibition or FOXO3a elevation could be a promising strategy for suppressing tumor metastasis.

The p53-related transcription factor p63 comprises two class of isoforms, TAp63 and ΔNp63. It has been demonstrated that TAp63 suppresses sarcoma development and mammary tumorigenesis in vivo [54, 55]. ΔNp63α, the predominant isoform of ΔNp63, drives SCC initiation and progression by promoting cell survival, proliferation, and stemness. Mechanistically, ΔNp63α transcriptionally activates pro-survival genes (e.g., FGFR2, EGFR) [56–58], and represses tumor suppressors (e.g., p73, p21) [59–61]. Additionally, ΔNp63α sustains cancer stem cell populations by upregulating stemness markers (e.g., SOX2, Nanog) [62–64]. A substantial body of evidence indicates that ΔNp63α functions as a critical metastasis-suppressing factor [13, 65, 66]. Notably, in contrast to our findings in this study, up-regulation of ΔNp63α has also been implicated in promoting EMT and tumor metastasis in certain contexts [64, 67]. These discrepancies may reflect differences between oncogenic and normal physiological signaling, depending on the cellular context.

ΔNp63α is tightly regulated at multiple levels, including transcription, mRNA stability, translational efficiency, post-translational modifications [10, 18]. Our previous study demonstrated that hypoxia-activated IRE1α-XBP1s-HDAC2-EZH2-H3K27me3 axis suppresses ΔNp63α expression at the epigenetic level [18]. In this study, we provide evidence that ATF6α inhibits ΔNp63α at the transcriptional level by promoting FOXO3a degradation, independent of H3K27me3, thereby expanding our understanding of distinct regulatory mechanisms by which UPR signaling modulates p63 expression. While this study elucidates a novel mechanism linking ER stress to breast cancer metastasis via the ATF6α-GRP78-AKT1-FOXO3a-ΔNp63α axis, several limitations should be acknowledged. First, our findings are predominantly derived from in vitro and in vivo models of triple-negative breast cancer (TNBC), specifically HCC1806 cells. Although these models are biologically relevant to ΔNp63α’s role in basal-like cancers, and our previous study had proved that activation of HER2 promotes tumor metastasis by suppressing ΔNp63α expression [14], the generalizability of our conclusions to luminal subtypes remains untested, where ΔNp63α expression is minimal and distinct regulatory mechanisms may dominate. Second, the tail vein metastasis model, though widely used to study lung colonization, bypasses early metastatic steps (e.g., invasion, intravasation) and may not fully recapitulate the natural metastatic cascade. Complementary orthotopic or spontaneous metastasis models could provide deeper insights into microenvironmental interactions. Despite these limitations, our work establishes a foundational framework for targeting ER stress pathways in TNBC metastasis and underscores the need for context-specific therapeutic strategies.

## Supplementary information

Supplementary Figures

Supplementary Table 1

Supplementary Table 2

Original Western blots

## Data Availability

The data analyzed during this study are included in this manuscript.
