# Supplementary material for: ATF6α inhibits ΔNp63α expression to promote breast cancer metastasis by the GRP78-AKT1-FOXO3a signaling
Source: Cell Death Dis. 2025 Apr 13;16(1):289. doi: 10.1038/s41419-025-07619-8 (PMC11994819; doi:10.1038/s41419-025-07619-8)
Supplement: Supplementary file 1 — Supplementary Figures [file 41419_2025_7619_MOESM1_ESM.docx]

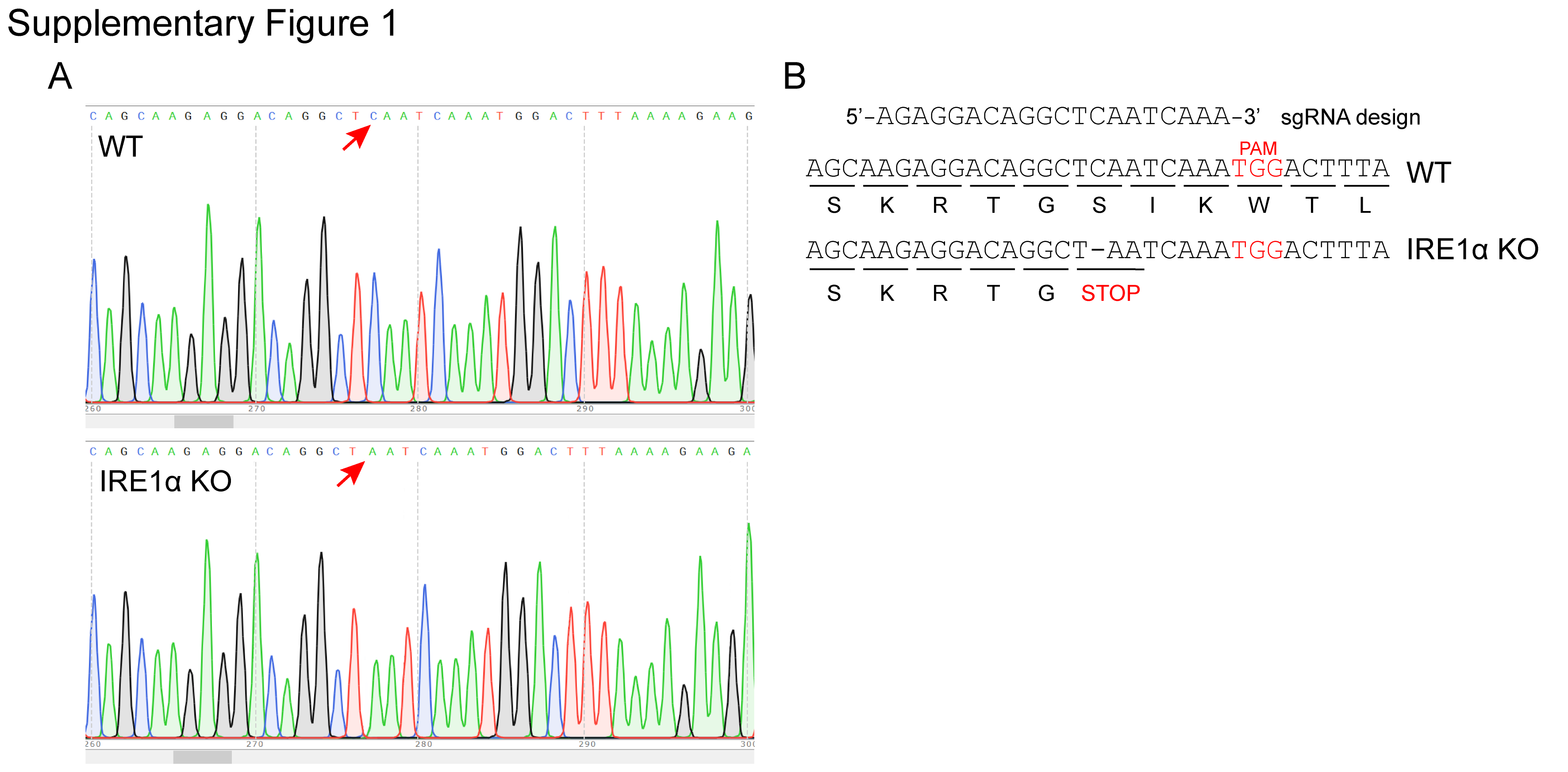


**Supplementary Figure 1 IRE1α KO cell was validated by DNA sequencing**

**(**A) HCC1806 cells (WT or IRE1α KO) were subjected to DNA sequencing. (B) DNA sequences were aligned.

**Supplementary Figure 2 The putative binding sites on the *ΔNp63* promoter**

>TP63 DNA sequence

CATTTTTACCCAGGGGGCAGCGTGACCCCTAATTCAGAGACCGTGGAGCTTTACGAAGTCTGAGGAAATGACTGAGAAAAGGCAAGAGCTCTAAATAGAGTTGTTCTAACTACTGAGGACTTGGGACATTGACGTAAAATCCTTGCATTTCTTTATTTATCTGAAAAATGTAGATAATAATACTAACTTACACCACTAGGTGGTTAGAAAATTAATGCATGTTACTAAACATTTAGTACATTTGAGATCCACATATAAGCATTGTTGTTGAGTGCAGAGAATTGTTACTTCTTTGTAACCTGCTTAAATCAATGCATGATATTTACATCAGCTACTCACTAATTCTGAAACCAAACATTATCAGGTATACTTTAATTAGCTCTTTTATGGAAATGTTTGTGCTGAGTTCAAATCTACATGAACTATTAGCGAATTTTTCATTGTGAGGATACCTTAAAGCAAAAAATATGGATTTTCCACCAATATGTTCCTTTTCCTTTATGTCATTTAAGTATCTTAAGTAGCTGTGCAGCTTGACTACTTCACTTGTACAATATGAAGATGAGCAAACTGAGGGACAAATAAATATCCGAGTAAAATCAGAGGAATATCCTCTAAATCACTTCTCTGATTCTGGACAAAAACAGGCTCTTATCTTTGTAATTGTCTTTAGAAATCATTTAGCTCTGCCCTCATTTCACAAATGAGGAATCTGAATCCAGAGTGGTTAAGTAACTTGCCCCAAGGGCACGGAGCTCCTGTCTCCTAACTCCCAGTCCAGTGCTCTTTTCTTCCAACATACTCAAAGTTCTCAAGTAGCCATAGTGGATATATTGTCTTAGATTTTGTCCAGAATCTTTGTAGATATGTTAGACAGAGACCGTATTTGGATTCATG

**P1**

CTGATAGCTGAGGTGGCACATGGAAGGAATCTGACTTTTTCATCTTTCAAATCTTGCTGGGAATCTTTTGACTTCGTGAAAGGTGAAGTTTGTTTACATTTTTAGTGGATATCAATACTTGGGACCCTGAGCCTTAGATTTAGGTGTTCAAATGGCTACATGATCCCATTAGTGTAAAAAACAATTTAATAATGACTTTGGTAGGCAGTTGTGCTAACAGCATTTCCTCTAGATCATTGATTCCCAAGCGTGACTCATCCAGAACCGCCTAGCAGGCTGTATAAAATCCAGTTGTTGGGGCTGTAACCCTGCAGAGTCTGATTCAGGAGGTCTGGGGCCACGTCCAGAAATCTGTTTTTAAAAAGCACCCTGGATGATTTTCAGGATCATCCAAGTTTTTGGGCCACAGGAATTATGCATACCTTTAAGGAAGTAACCATCTGCTTTCTACTGTTGACTCTTAAGCTGTCAGTAGGTGTAGAATTTAGACACATTGTACAAAAAATTATACTGTATTTTGTAAGTAGGTTTTTTTTTTTTTGCCTTCTAGGCAGTGCTCATTTGGTTAATTATAGGTAGAAAGAGAGATCATTCTTCCCAATATATGTGTGAAGAAATGAATGTTTTGTCTGATATAAATTTGTAGTGTTACAGAGTACTGTCATATTCTTGCATACTCAAGATCAAATTATATTATGGGAGTTTTTTGGTTTTTGTGTATAAAAGAAGGAAGCATTATTACCAGTAACCTATGGGTCT

**P2**

TATATATCAGCCAATGACCACGTCATCCTTTAAGTGCATAAATTTTATGGATGCAAAAGTAGAAGTAAAGGGGAGGCAAATCCGAAGTCGTGAACGTATTTGCAAACTTTGTTTCTGAAATGTATGTTCTAATCTCTTCTGGCTTCCAATTCTCTTCTAAACTTTGTGTCTTCTAAATTTTAGAAGAAATCACAATTTTCACTTATAGTTATCTTGGCCACTTACAACCTTTCAAGCAAAATAGAGGAAATTGGGTCCCTGAGTGGGGGGTGGGGGTGTAGGATGATGTCAAGAAATAAATCTAACTGAATTACAAATACTCAGCAGTAAAGAGAGTATTTTAATGAATCAGATGATAACCATCAGGGTGCATTTCATCTCCACTAAATCTTCGTACCAAGGCCAGATTCTACATGAATGTTGGTACGTATTTATGTAAATGTATTTTTAAAACAAAAGCCAATTGATATCTTATGCTTTAATACTTATTCCACTAATCATTTACATAGATGCATCACGTGCAGTAATCATTTTTATTACCTATTCACAAGCAAAAATATTAGTATAAACTGGGTACAATAAAATAGAGAAAAGAAATATTCAAATAGATAAGCGTTTTGTTAAAAAAAAAAAAAAGAAGAAAGAAAGGACACATTTATCAGGATTCCTATTTCCCGTACATAATATGGA

**P3**

TGTTTGTTTGTTTTTGTAAGTTAACGGGACCGGTGGTTTAACTTGTTATTGAAACATGCTCGAAAAAATCAGGTAGCTTATTTTGTAATTGCTTGTTATGAAACCACTGGCATTTCTCTGGGGAAAATAAGTTAAAAACTCTTTAGCTATCAGGCAGTGGGTTTTAATTTTTTATATTGGTTAAATGTAACAGTGGATTTGCGTACTCTCTCCTAATTTCTAACTTTGTGTAATCATTCTTGAAACCCCAAATCTAGATTTTAAAAAAGAAGCCTTCTAAAAGTTTTCCTGAAGTTTACTTTTCAGTTACAAAGAGTAAAATAACTTTCTGAAATGCCTTCTGTAAATCGTGGTGGTGGTGCGGTTTGTTTGGGGAGATTTGTTTTGTTTTTAAAAGACAGTGCACTTTCTTATGAAAGAGACAGGGAAAGTTTTACCTGTCTGTCTCCTGGGTTTGTTTTTTTTTTCTTTCTTTCTTTTTCTTTTAAAGATTGGTGATAAGGAATTCTAACTACTTAATGAGATGGGAGAGGCCTCACTCCATTGGAGTGGAGGAGTCCAGGTGGAAGTTGATGGATTGGACAGGTAAAGAGAAGAGTCCCGCCTCCTCATGCCTATAGT

**+1 DNp63 TSS**

TGGGTATATATTAGGAAACCTTAAATTATGTAC**AGAGAGAGAAAGAGAGAGAGGGACTTGAGTTCTGTTATCTTCTTAAGTAGATTCATATTGTAAGGGTCTCGGGGTGGGGGGGTTGGCAAAATCCTGGAGCCAGAAGAAAGGACAGCAGCATTGATCAATCTTACAGCTAACATGTTGTACCTGGAAAACAATGCCCAGACTCAATTTAGTGAG**GTAAGGATTTTAGATTTTAGCACTCCATTTAGAGATGCTTTTTAATTTTTATTTTTGTAAAAAAACTTACGTATTTGCGGTTCTCGGTCACCCAATGTAATGTTTTGCAAATTGTATATAGGAATCTCCTTTTCTTGGTTAATGTTTTCTGTGGTGGCTGTAAGATTTTTTTTTTTTTATTAAGTAGGAGATGAAACAGTAGGAGAAGATGAAAAAGAAAATCTGGGTGACATTATGTATTTGAAAAAATAATATTCAGGAGTTTATAAAATCACTTTTTAGAAAAACAGACTCAGAGAGCAGATCACACTCTGCTAGATACATTGCAATGAATCCTCTGATGGGTATTCATGTACCAGTAAGAAAAATCAGTTGACTGTGTCTTAACTTCTTAGCATAGTTCTATTTCCAGACTTCATCCTAAAAGCAAATGAAGCTTTTTTCAAAATAATCAGGTGAGATTTTTTAAAGCCATCTTCGTTTTTAAGTCTGTCAATCCAGAGACCCACCTAAATCCAGAAGGGGCTGTGATAACGTTTCTGTCGTCTGGAGGGTGACTTGTACTTTCACGTGTGATGACAAATAAAGTTATTTTGGAGGGATGCATTGTTAAATATTTATACATGCATACATGTAAATGTGTATTATAGCATACAGGATTTTATTTAAGAAACTGATTATTTGCATTTCTGATCTCTTCTCAGCAAGTTTGTCTTAGGTATTTAAACCTGTGCAAAGGGATAAAAAAAAAAATCCTGAAGAGACTGTAAGGCTTTGAGGAAGTTTCCTAAGGTTGAGGGAGGTCTAACAACAATATTA

**P4**

GTTTACATTCCTCAGAATGGGGCAGCTGTGTTGACACTAATCAGATTGGGTTGTGGTGGGGAGTGTTGTGGAGAGAGAGAGTTCGGTGTTTCAGATAAAAATGCTTGCAGGTGAGTGCTTGGAAAAGTGGACTAGAGTACTCAAATTGAGCTCCTTAAAAATCAAAACAAAATGCATAGTATTCCGGTGCAGACCGCAAGAGGAAGAGTAGAGCAGAGGGCTGCGAAGCAGGGCCTGTGCTATCGTATTGACCACGCTATTGTCTAGACTCCTGAACTGTGTAAATACAACAGGGGAAATATCGAGTTATCTAAAGAAGGAAAAGGAAGTGCCTATTTTTCTTTAAAACATTCAAGCAGGCTTGCTTGTCCTCAGTAGAGAAACTTGAGTTTCTTGTTTTGCAGTTATATTGAAAGTTAGCTTAAAATGTTACCAATTTGTTTTTAAATAGGATTAGCTTTTAAGATCTTCTTGATTCTATTGGATCCAATTTTGTCCAGTTTTAATCATTCAGAAAATGATCTGTCCATCATGTCATACCTGAGTTTGTTTGTCTTTATTCATGAACTATTTCACACACAGTATTTATAAGCCGCCTAGGATCTTGTAAGTCAAATGGGAGAAGCAAAAGTCTGTTCATTGATATCTCTAAGTGAAGCACAAGGTTGATGTAAAGTGGCAAGAAATGATTAATTTTAGGTTAAATATAATGTCGTGAAAGAAAGCAATTGCATTAATGTTGTGATTAGAGGATATTTGTAGAAATTATTTCCTTTTATGGAGGCAAAAGAGTCATATTGTATGTGAGAATATGGCATGCTTATGAAAGAAAATAGTTTTATACACAGGTTTTAGTTTTTCACAACTCTAGGTAAATATGTAATCAAGAGCTACAAAAGGCATCCCTTGTCTGTTTTCTATTGGAATGCTGCTAAGGTCGACAAAAAATATCTGTAAAAAACATGGCTGCACTTCCCTACCTTTCTATGTCTTTTTGCATGTTGCCTGCATGACTTTCATATCCTTCCACCAGTGCATGTATTAGGTTATAATTTCATGGCTTGACTTCCATTCTATTACGTACGAACAACTTTCTTTCTGTACAGGATTATGCTTTCATATCTAGTCCTCTGTTAAAATTCAGTGCTACATCTGAGTATATGAAATGCCTGGCACCACATGGCTCTGAGAACCATGAATTTAATATAAAAGTTATAATGTATAGGGATTTATGTTTATTAGTAACTGTCAATACAGAAAAAGCAATCTACTATTATTACACAAGAATTGTTAAACATTGGGTAGATTAAAAATGAAAAAGGCATAAAACTCCCTGTAAGAGTGAAAATTATTTAAAAACTATAATCCATTCTTTGACTTCAAATAAATTTATAGTTTAATTTGCTAGCAATTTCTTTGAGTTGATAACTCAAAAACTATATAAGAACACTGTTCCTTCTCTATTCCTTTTCCCACAACTTTTGAATTCTTCCCCAACCCTTAGCAGATTTGGATTTGGGGGAGCTGCTAATTAACAGGAAATGGTCCGGAAGGGAGGAGAACACAAGCAGGATATAACAGGCAAGGCTGAAGGCTCTACCCTTGGGTACAGCGGAGAGGCCGCAAGCAAGCTCCACTCCCCAGTGCCAGACTAGGAAAGACAGAGCCTCTGGGAATGGTCTCAGACTTGGAGATAGAGAGGGTCAAAATAAAGGAACTTCTTCACAGATCAAGTTTTAACTTCAACTTTCTGGCAAAACAAAACAAAACAAAACTGGGCTGTGTGTGTGGTGTAAGTGTGTATGTGTGTGAGTGGGGAGGATCTCAAACTCCTTCTTAGAAAGAGATATTTACTAAAATTCCTCATCCGGGGTTTTAGATGATGAAGGAAAGACACTTTGGTAAATGGGTTACAGTATTTTCGAACTAAATGGGAAATCAAGGTTCATATATGGAAACACCTCATTTTACAAATCAGGGTTAATGATTTTTCTTTCT


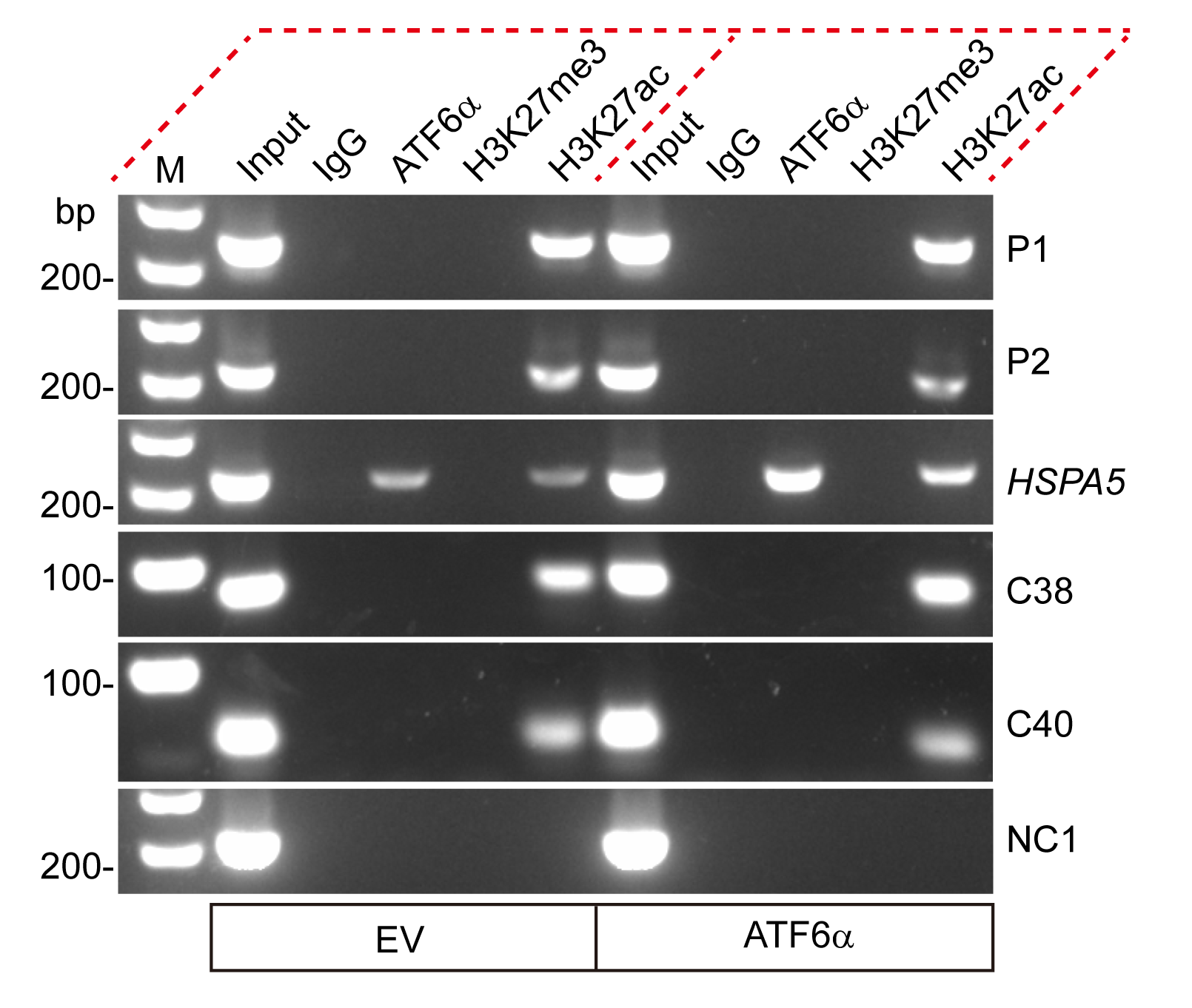


**Supplementary Figure 3** ChIP assays were performed in HCC1806 cells (EV or ATF6α) by using indicated antibodies or a normal rabbit IgG. Primers specific for P1, P2, HSPA5, C38 or C40 were used for PCR; a randomly chosen segment (-516 to -314) was used as control (NC1).


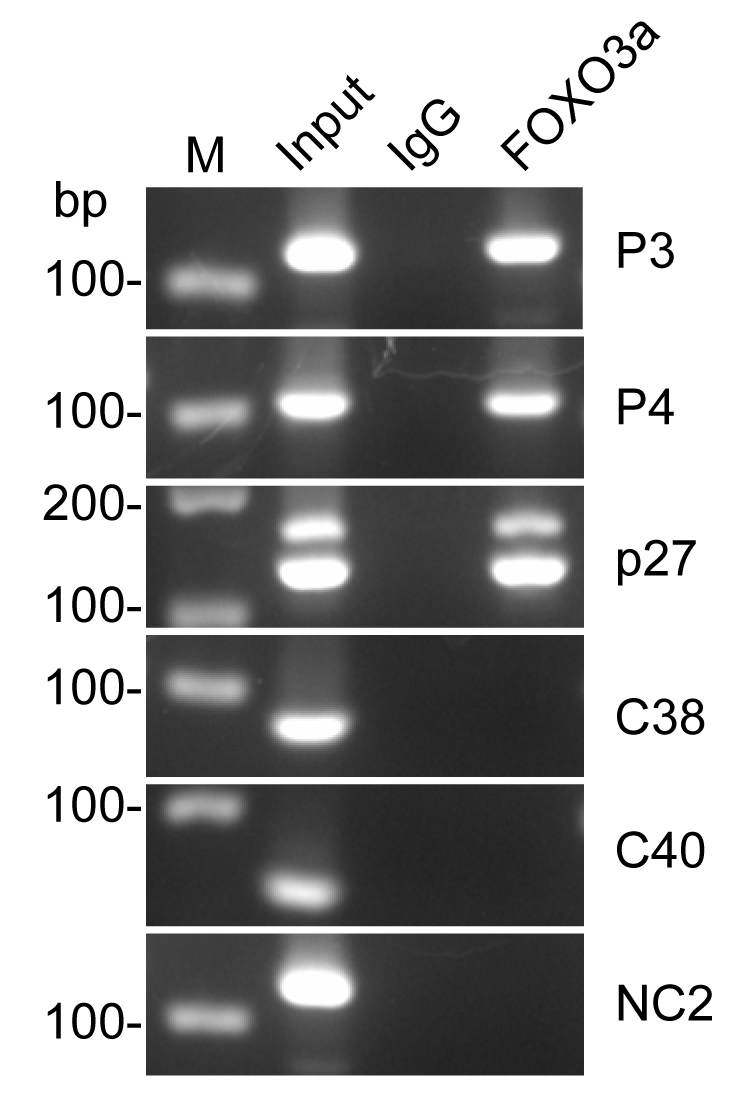


**Supplementary Figure 4** ChIP assays were performed in HCC1806 cells using a specific FOXO3a antibody or a control rabbit normal IgG; a randomly chosen segment (−469 to −362) was used as control (NC2).
