# Supplementary material for: ATF6α inhibits ΔNp63α expression to promote breast cancer metastasis by the GRP78-AKT1-FOXO3a signaling
Source: Cell Death Dis. 2025 Apr 13;16(1):289. doi: 10.1038/s41419-025-07619-8 (PMC11994819; doi:10.1038/s41419-025-07619-8)
Supplement: Supplementary file 3 — Supplementary Table 2 [file 41419_2025_7619_MOESM3_ESM.docx]

**Supplementary Table 2 Primers for ChIP-qPCR**

ChIP-P1-F：GTTCTCAAGTAGCCATAGTG

ChIP-P1-R：GGCTCAGGGTCCCAAGTAT

ChIP-P2-F：CTTGCATACTCAAGATCAAA

ChIP-P2-R：AGAACATACATTTCAGAAAC

ChIP-P3-F：GAAAGAAAGGACACATTTATCAGG

ChIP-P3-R：CCTGATTTTTTCGAGCATGTTTC

ChIP-P4-F：TTCCTAAGGTTGAGGGAGGTC

ChIP-P4-R：ACAACACTCCCCACCACAAC

ChIP-HSPA5-F：CGCAGGAGAGATAGACAGCT

ChIP-HSPA5-R：GTGAAGGCCCTGCTCGTTGG

ChIP-p27-F：GTCTGTGTCTTTTGGCTCCG

ChIP-p27-R：CGTTAGACACTCGCACGTTT

ChIP-NC1-F：CTCTTTAGCTATCAGGCAGT

ChIP-NC1-R：CGATTTACAGAAGGCATTTC

ChIP-NC2-F：TGTAACAGTGGATTTGCGTAC

ChIP-NC2-R：TCAGGAAAACTTTTAGAAGG
