# Supplementary material for: ATF6α inhibits ΔNp63α expression to promote breast cancer metastasis by the GRP78-AKT1-FOXO3a signaling
Source: Cell Death Dis. 2025 Apr 13;16(1):289. doi: 10.1038/s41419-025-07619-8 (PMC11994819; doi:10.1038/s41419-025-07619-8)

Figure 1

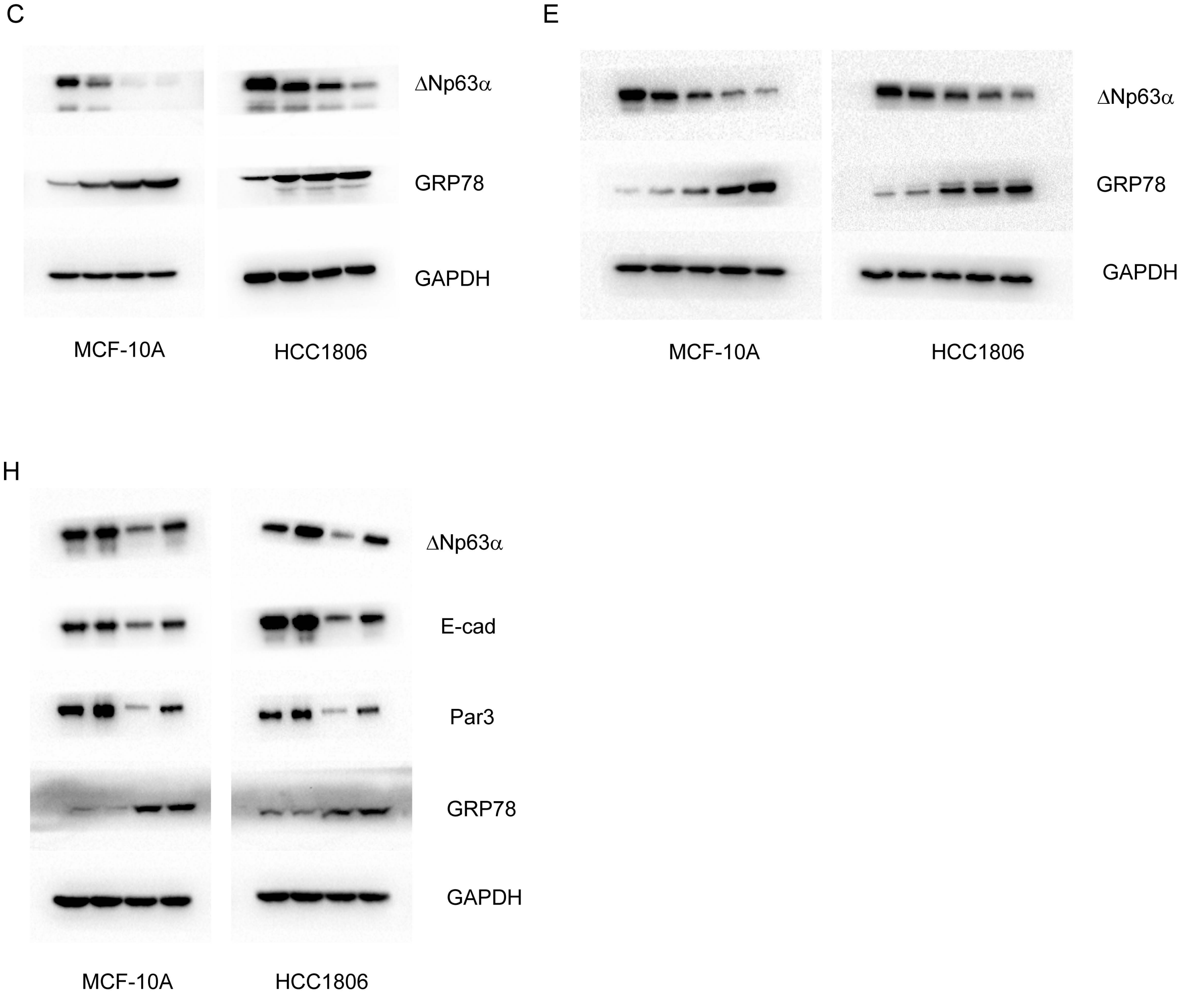

C
ΔNp63α
GRP78
GAPDH
MCF-10A
HCC1806
E
ΔNp63α
GRP78
GAPDH
MCF-10A
HCC1806
H
ΔNp63α
E-cad
Par3
GRP78
GAPDH
MCF-10A
HCC1806

Figure 2

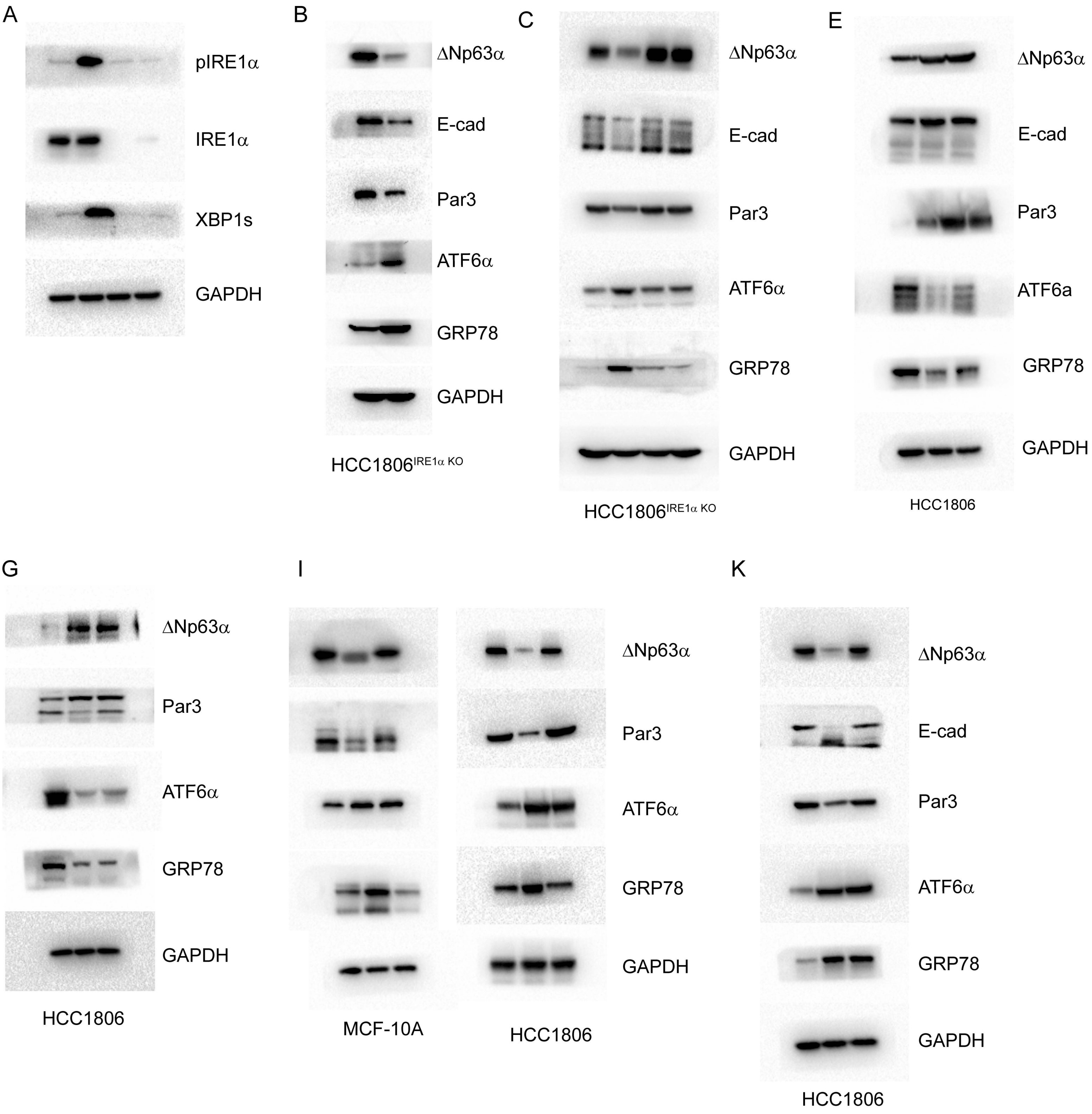
A
pIRE1α
IRE1α
XBP1s
GAPDH
B
ΔNp63α
E-cad
Par3
ATF6α
GRP78
GAPDH
HCC1806IRE1α KO
C
ΔNp63α
E-cad
Par3
ATF6α
GRP78
GAPDH
HCC1806IRE1α KO
E
ΔNp63α
E-cad
Par3
ATF6a
GRP78
GAPDH
HCC1806
G
ΔNp63α
Par3
ATF6α
GRP78
GAPDH
HCC1806
I
ΔNp63α
Par3
ATF6α
GRP78
GAPDH
MCF-10A
HCC1806
K
ΔNp63α
E-cad
Par3
ATF6α
GRP78
GAPDH
HCC1806

Figure 3

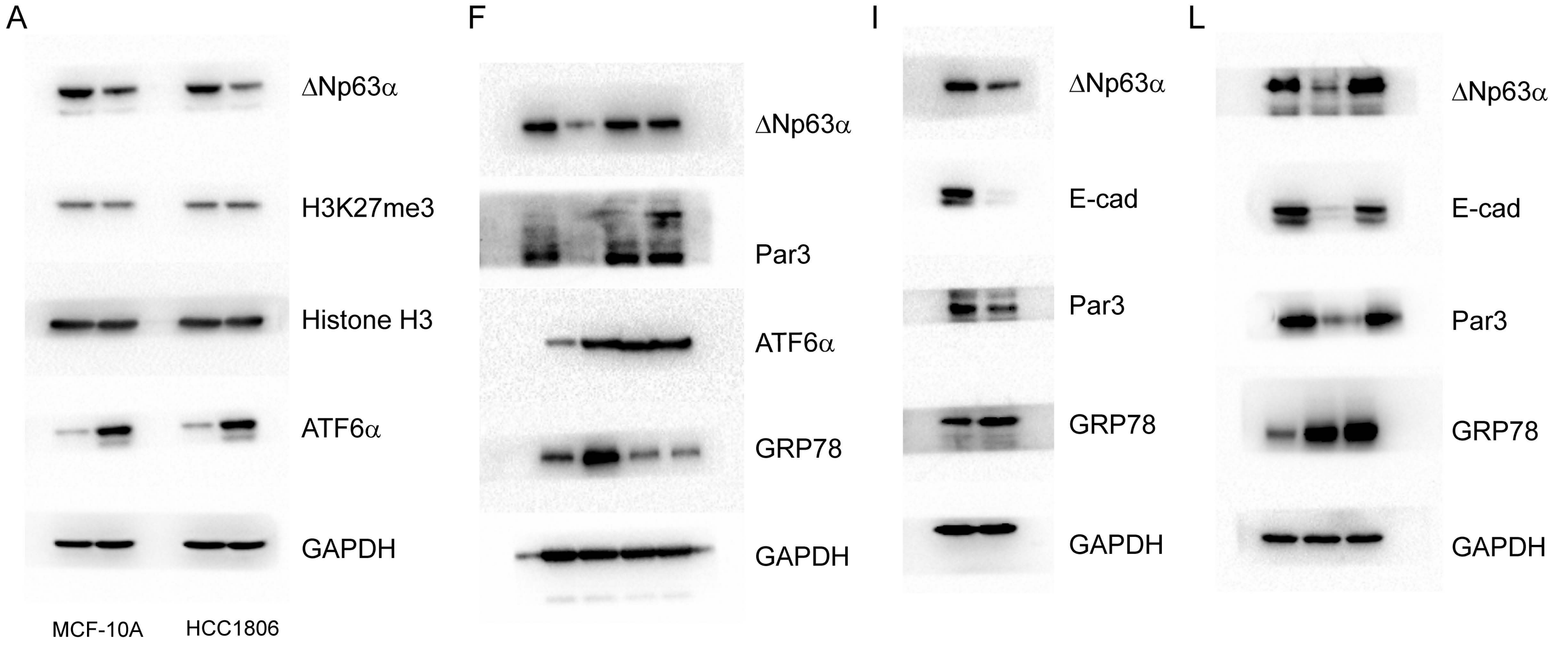
A
ΔNp63α
H3K27me3
Histone H3
ATF6α
GAPDH
MCF-10A
HCC1806
F
ΔNp63α
Par3
ATF6α
GRP78
GAPDH
I
ΔNp63α
E-cad
Par3
GRP78
GAPDH
L
ΔNp63α
E-cad
Par3
GRP78
GAPDH

Figure 4

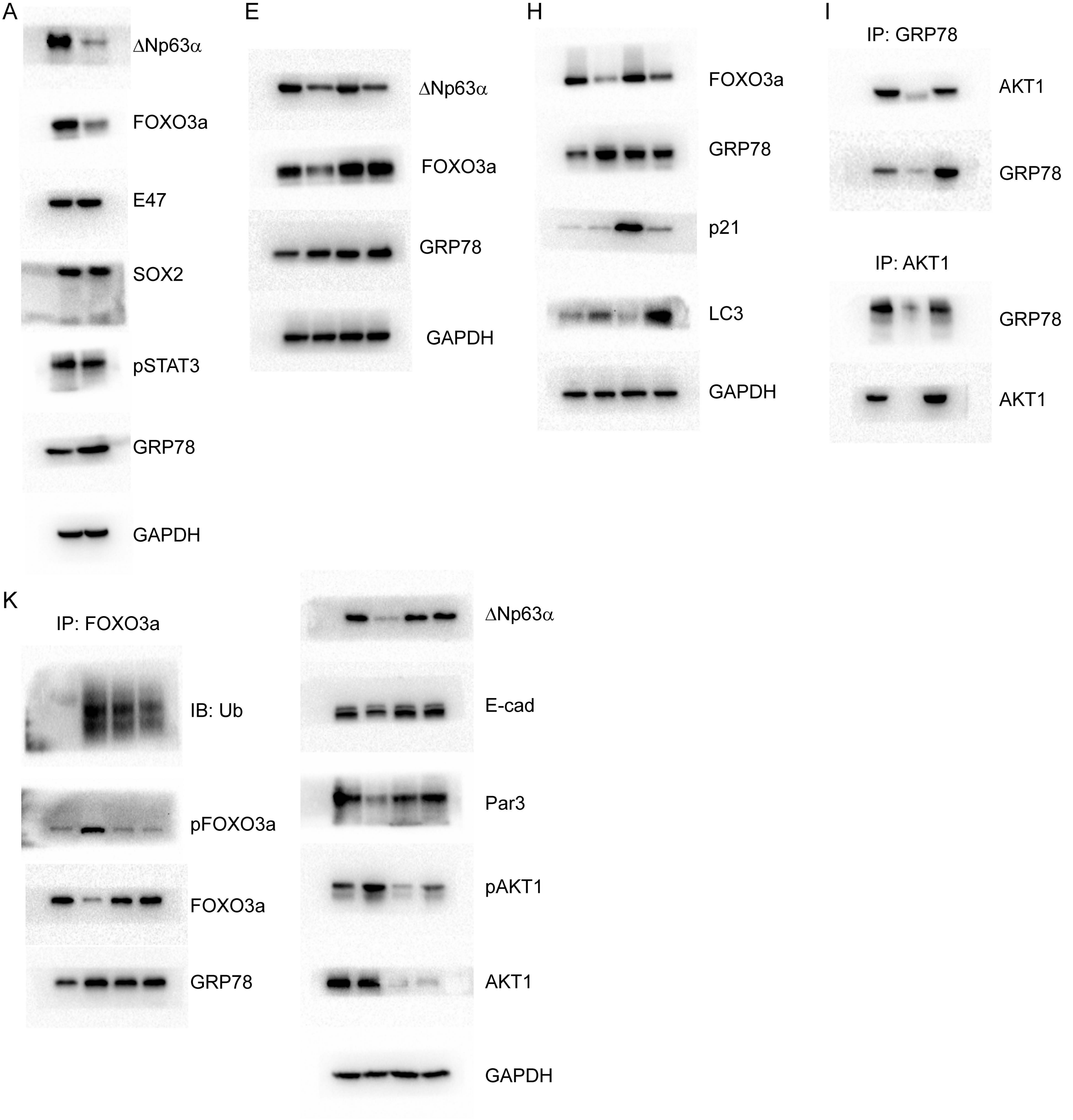
A
ΔNp63α
FOXO3a
E47
SOX2
pSTAT3
GRP78
GAPDH
E
ΔNp63α
FOXO3a
GRP78
GAPDH
H
FOXO3a
GRP78
p21
LC3
GAPDH
I
IP: GRP78
AKT1
GRP78
IP: AKT1
GRP78
AKT1
K
IP: FOXO3a
IB: Ub
pFOXO3a
FOXO3a
GRP78
ΔNp63α
E-cad
Par3
pAKT1
AKT1
GAPDH

Figure 5

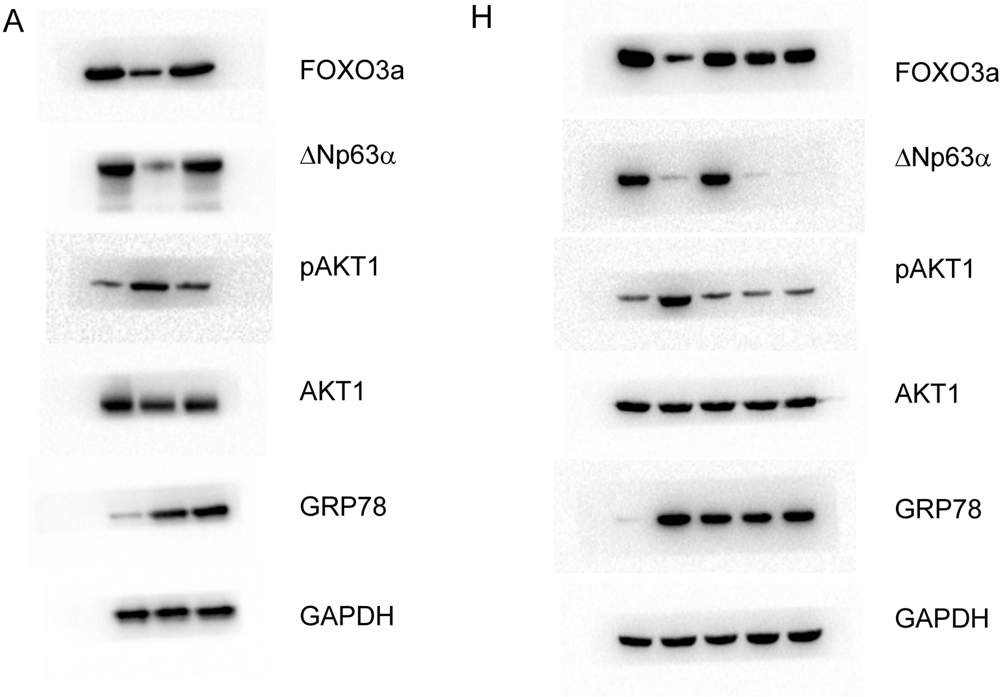
A
H
FOXO3a
ΔNp63α
pAKT1
AKT1
GRP78
GAPDH
FOXO3a
ΔNp63α
pAKT1
AKT1
GRP78
GAPDH

Supplementary Figure 3

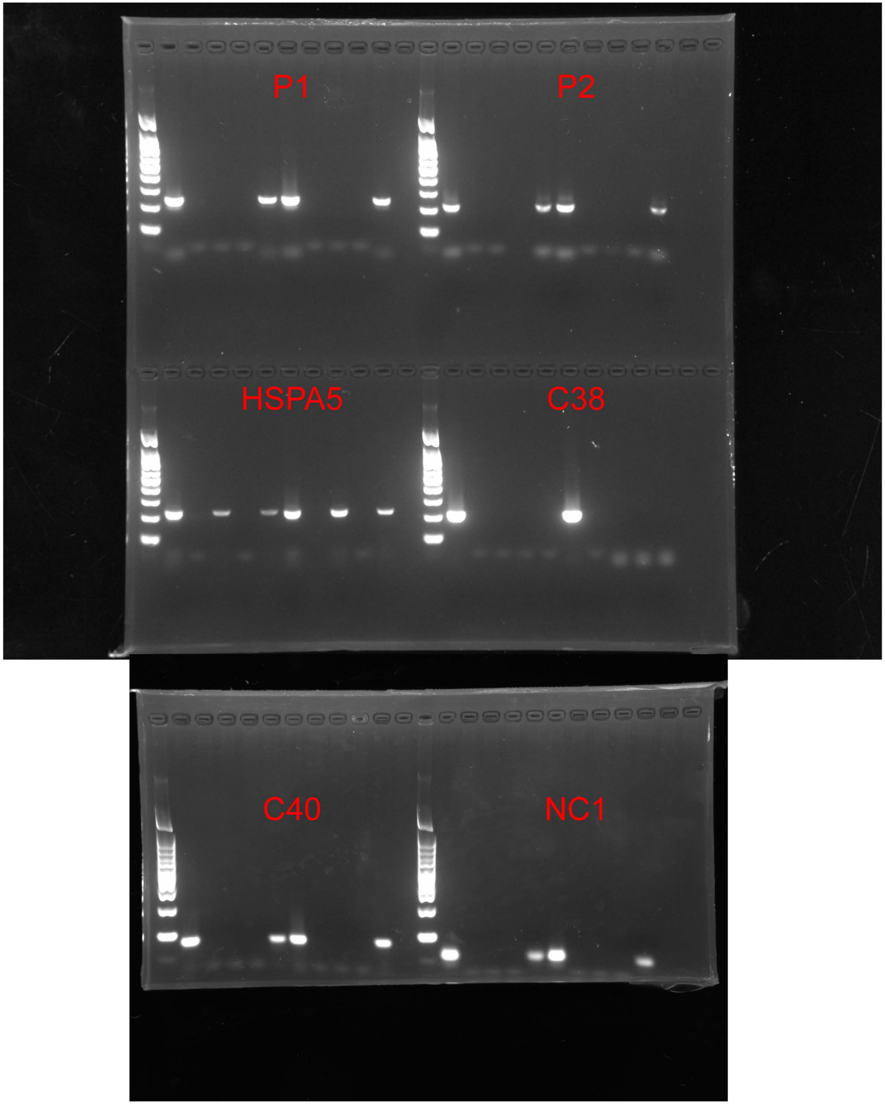

Supplementary Figure 4

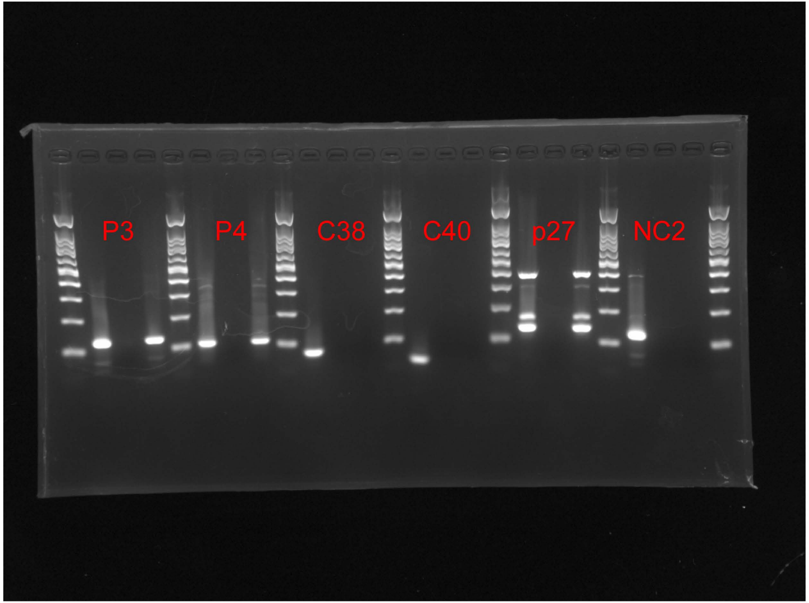

Supplement: Supplementary file 4 — Original Western blots [file 41419_2025_7619_MOESM4_ESM.pdf]
